# Unsaponifiable fraction of black *Vitis vinifera* seed oil attenuates liver cancer progression by targeting apoptosis and key tumor-associated genes: In vitro, in vivo, and in silico studies

**DOI:** 10.1038/s41598-026-44404-9

**Published:** 2026-04-10

**Authors:** Marwa M. Abu-Serie, Ashraf A. El-Faham, Ghada M. Ahmad, Noha H. Habashy

**Affiliations:** 1 Department of Medical Biotechnology, Genetic Engineering, and Biotechnology Research Institute, City of Scientific Research and Technological Applications (SRTA-City), New Borg El Arab, Alexandria, 21934 Egypt; 2 Biochemistry Department, Faculty of Science, Alexandria University, Alexandria, Egypt; 3 Department of Medical Laboratory Technology, Faculty of Applied Health Sciences Technology, Pharos University in Alexandria, Canal El Mahmoudia Street, Beside Green Plaza Complex 21648, Alexandria, Egypt

**Keywords:** Black *Vitis vinifera* seed oil, Unsaponifiable fraction, Anti-hepatocellular carcinoma, Tumor-associated genes, Apoptosis, Biochemistry, Cancer, Cell biology, Drug discovery, Plant sciences

## Abstract

Cancer is the leading cause of death worldwide. This study evaluated the anti-liver cancer influence of the unsaponifiable fraction (UnSap) derived from black *Vitis vinifera* seed oil (BVVO) using the HepG-2 cell line and a HCC mouse model. Compared with 5-fluorouracil (5-FU), UnSap exhibited a strong antioxidant capacity, excellent safety (in PBMCs), and a higher selectivity index (SI) with a lower IC₅₀ against HepG-2 cells. It effectively induced apoptosis and modulated the expression of key genes involved in cell cycle progression. The docking results predicted that the abundant constituents of UnSap can interfere with the interaction between β-catenin and human T-cell factor-4 (HTCF-4) and may inhibit the activity of key regulatory proteins involved in HCC proliferation and stemness. In HCC-bearing mice, UnSap significantly improved the expression of serum liver function markers (0.4–1.3-fold), restored redox balance (> threefold), and preserved hepatic tissue architecture. It also modulated the expression of critical genes associated with apoptosis (> 20-fold), cell cycle regulation (> fourfold), inflammation (> fivefold), angiogenesis (threefold), and cancer stemness (> 45-fold). These therapeutic effects are likely attributed to its high phenolic and phytosterol content, as confirmed by HPLC analysis. Across all investigated parameters, UnSap demonstrated superior anticancer efficacy compared with 5-FU. These findings highlight the potential of UnSap as a promising natural therapeutic candidate and provide a foundation for future studies on its bioactive constituents for the development of novel anticancer agents.

**Supplementary Information:**

The online version contains supplementary material available at 10.1038/s41598-026-44404-9.

## Introduction

Hepatocellular carcinoma (HCC) is one of the most prevalent cancers worldwide. It is a significant cause of cancer-related deaths, particularly in regions where aflatoxin and hepatitis infections are predominant^1,2^. Approximately 85% of HCC cases occur in developing countries^3^. In Egypt, HCC is the fourth most common cancer. The hepatitis C virus (HCV) has had a significant impact on the country, leading to more than 63% of all HCC cases^3^. To combat HCV, the government launched the "100 Million Healthy Lives" campaign in 2018, which reduced HCV incidence to 4.6%^4^. However, the annual rate of HCC among patients with cirrhosis receiving direct-acting antiviral therapy remains high at 29 cases per 1,000 patients^5^. In addition, HCC in Egypt is related to other conditions, such as alcohol consumption (12%), HBV (13%), and other causes (12%), as well as alcoholic and non-alcoholic cirrhosis^3^.

HCC development is a multistep process involving cellular and molecular changes stimulated by diverse endogenous and exogenous stimuli. One type of endogenous damage arising from the cumulative production of oxygen (such as superoxide anions and hydroxyl radicals) and nitrogen-free radicals (e.g., nitric oxide) is termed oxidative stress^6^. Oxidative stress induces a cellular redox imbalance, which has been found in various cancer cells, including HCC cells, compared with normal cells; this redox imbalance may thus be related to oncogenic stimulation. Free radicals attack not only the bases but also the backbone of both mitochondrial and nuclear DNA. Endogenous DNA lesions are genotoxic and induce mutations; therefore, they are potential biomarkers of carcinogenesis. Thus, free radicals are considered an essential class of carcinogens^7^. Furthermore, free radicals in cancer cells are involved in all stages of HCC progression. The effects of free radicals are balanced or scavenged by the antioxidant action of enzymatic and non-enzymatic antioxidants^8^.

Owing to the late diagnosis of HCC, lack of available effective treatments, and high rate of resistance to common chemotherapies such as 5-fluorouracil (5-FU) and sorafenib, it presents management challenges^9^. This emphasizes the importance of developing novel therapies that are safer, more effective, and have fewer adverse effects, especially those derived from natural sources.

For a long time, natural products have been a key source of bioactive compounds for cancer drugs. Many plant-derived anticancer drugs can trigger apoptosis, stop cell growth, suppress new blood vessel formation, and reduce oxidative stress^10,11^. Among these, the seeds of *Vitis vinifera* (VV, grape), especially those of black grape varieties, are rich in phytochemicals, such as flavonoids, phenolic acids, and phytosterols. These compounds are known for their antioxidant, anti-inflammatory, and anticancer effects^12,13^. While most studies have focused on the polyphenolic components of grape seed extracts, the unsaponifiable fraction (UnSap), which contains valuable lipophilic compounds such as phytosterols, tocopherols, and triterpenes, has received less attention, especially in the context of liver cancer. New evidence suggests that UnSap components may have anti-proliferative and pro-apoptotic effects in different cancer types^14,15^. Additionally, phytosterols, such as β-sitosterol, can influence oncogenic signaling pathways, including the nuclear factor-kappa B (NF-κB), protein kinase B (AKT), and TP53 pathways^16^.

In this study, we evaluated the anticancer effects of UnSap extracted from black VV seed oil (BVVO) against liver cancer. We analyzed the chemical composition, which includes phenolics and phytosterols, along with the antioxidant potential of BVVO-UnSap via high-performance liquid chromatography (HPLC) and radical scavenging assays. We used both the HepG-2 cell line and a mouse model of *p*-dimethylaminobenzaldehyde (*p*-DAB)-induced HCC. Our goal was to examine how BVVO-UnSap modulates apoptosis- and cell cycle–regulating genes in HepG-2 cells. These genes included tumor protein P53 (*TP53*), BCL-2-associated X protein (*BAX*), retinoblastoma (*RB1*), cyclin-dependent kinase inhibitor 1 (*CDKN1A*), E2 promoter-binding factor 4 (*E2F4*), Kirsten rat sarcoma viral oncogene (*KRAS*), and B-cell lymphoma 2 (*BCL2*). HepG2 cells were chosen for the in vitro study because they are a well-characterized HCC model and are commonly used for mechanistic investigations of proliferation and cancer stemness. Docking analysis was used to predict the ability of the abundant constituents of UnSap to interfere with the interaction between β-catenin and human T-cell factor-4 (HTCF-4). In addition, it is expected that these ingredients will inhibit the activity of the epidermal growth factor receptor kinase domain (EGFR), the NADPH binding domain of NADPH oxidase 2 (NOX2), and the smoothened receptor (SMO). To the best of our knowledge, this study is the first to evaluate the predicted mechanisms of these constituents on these cancer-related proteins. In mice with HCC, we evaluated the impact of BVVO-UnSap on liver function biomarkers, cellular redox state, liver tissue architecture, and expression of critical tumor-related genes involved in apoptosis, cell proliferation, stemness, inflammation, and angiogenesis. This work presents the first comprehensive evaluation of BVVO-UnSap as a therapeutic strategy for HCC. These findings support further investigations into the bioactive components of UnSap for the development of new anticancer agents.

## Materials and methods

### Chemicals and cells

Diphenyl-α-picrylhydrazyl (DPPH), Ficoll-hypaque, 5-FU, 3-(4,5-dimethylthiazol-2yl)-2,5-diphenyl tetrazolium bromide (MTT), standard phenolics, dimethyl sulfoxide (DMSO), tetramethoxy propane (TMP), 5,5'-dithiobis-(2-nitrobenzoic acid) (DTNB, Elman’s reagent), *p*-DAB, phenobarbital (PB), thiobarbituric acid (TBA), and reduced glutathione (GSH) were supplied by Sigma‒Aldrich (St. Louis, MO, USA). Ascorbic acid (Asc) was purchased from Riedel-de-Haën (Germany). The GeneJET RNA purification kit, 2X SYBR Green master mix kit, and cDNA synthesis kit were obtained from Thermo Fisher Scientific (Fremont, CA, USA). The HepG-2 cell line was obtained from the American Type Culture Collection (ATCC, Manassas, VA, USA). Fetal bovine serum (FBS) and Roswell Park Memorial Institute (RPMI)-1640 medium were obtained from Lonza (Basel, Switzerland). The Caspase-Glo 3/7 assay kit was purchased from Promega (Madison, WI, USA). Albumin, total protein, aspartate aminotransferase (AST), alanine aminotransferase (ALT), alkaline phosphatase (ALP), and gamma-glutamyl transferase (γ-GGT) assay kits were purchased from BioSystems S.A. (Costa Brava 30, Barcelona, Spain). Forward and reverse primers were purchased from Bioneer (Daejeon, South Korea). Other chemicals were of high grade.

### Preparation of BVVO and UnSap

BVV (NCBI: txid29760) was obtained from a local cultivar in Wadi El-Natroun, El-Beheira, Egypt. The plant material was identified based on the characteristic macroscopic features of grape fruits described in standard botanical references and common horticultural classifications^17^. The fraction was prepared as previously described method^18^. The fruit skin and pulp were separated manually, then the seeds were washed thoroughly with tap water, air-dried at room temperature (25–30 °C), and ground. Crude BVVO was extracted using a Soxhlet apparatus with hexane at a 1:5 (w/v) ratio for 3.5 h. To obtain the UnSap fraction, 1 mL of BVVO was saponified with 20 mL of 10% methanolic KOH while heating at 80 °C for 3 h. The resulting aqueous UnSap fraction was then extracted using hexane. Hexane was then evaporated from BVVO and UnSap under reduced pressure using a rotary evaporator until a constant weight was achieved to minimize solvent traces. The samples were then weighed and stored at 4°C for further analysis.

To ensure the reproducibility and consistency of UnSap extraction, all batches were prepared using the same standardized technique under controlled conditions, such as fixed plant material-to-solvent ratios, extraction duration, temperature, and storage conditions. Batch-to-batch variability was measured by comparing the extraction yield, physicochemical properties, and total antioxidant capacity of each batch. Only batches with comparable antioxidant activity and acceptable variance limits were used in the following experiments.

### HPLC analysis of the phenolic and phytosterol constituents of UnSap

An Agilent Infinity 1260 HPLC instrument (Germany) equipped with an Agilent multiwavelength UV detector and fluorescence detector was used to detect phenolic, phytosterol, and tocopherol compounds. Gradient elution mobile phases A (0.1% acetic acid in water) and B (acetonitrile) at a rate of 1 mL/min were used for the detection of phenolics. The gradient program started at 90% A, increased linearly to 50% A over 20 min, then to 30% A over 10 min, followed by a return to 90% A over 5 min for column equilibration. The separation was done on a reverse-phase C18 column at 284 nm.

For phytosterols and tocopherol separation, isocratic elution was performed using acetonitrile and water containing phosphoric acid (0.1%) at a rate of 1 mL/min. The separation was performed on a Hyperclone reverse-phase column with a UV (205 nm, for phytosterols) and fluorescence detector (excitation 290 nm/ emission 330 nm, for tocopherols). The structures of the phenolic, phytosterol, and tocopherol ingredients of UnSap were obtained from the PubChem database (https://pubchem.ncbi.nlm.nih.gov/).

### Antioxidant activities

Different antioxidant methods, including total antioxidant capacity (TAC), anti-DPPH, NO, hydroxyl, and superoxide radical assays, as well as anti-lipid peroxidation and reducing power assays, were investigated. Asc was used as a standard antioxidant to compare the efficiency of BVVO and UnSap. By graphing the relationship between the percentage of radical or lipid peroxide scavenging and the log concentrations of the extracts or Asc, the IC_50_ value (or 50% inhibitory concentration) was determined. To observe the reducing power, a graph of absorbance vs. log concentration was plotted to determine the IC_50_ value (the concentration of extract or Asc that produced an absorbance of 0.5).

For the TAC method, 100 μL of BVVO, UnSap, or various concentrations of Asc (0–1 mg/mL) were mixed with 1.9 mL of the reagent solution (28 mM sodium phosphate, 4 mM ammonium molybdate, and 0.6 M H_2_SO_4_). The mixtures were incubated for 90 min at 95 °C, and the absorbance was recorded at 695 nm^20^.

The modified standard method of Blois^21^ was used to evaluate the DPPH scavenging activity of the studied samples by measuring the absorbance of the non-scavenging DPPH at 490 nm. The Griess reaction^22^ using Griess reagent (0.1% naphthylethylenediamine dihydrochloride, 1% sulfanilamide, and 2% phosphoric acid) and sodium nitroprusside was utilized to assess NO scavenging activity. A bright reddish-purple azo dye was obtained from this reaction, and its absorbance was measured at 490 nm. The ability of the studied extracts to scavenge hydroxyl radicals was assessed via a salicylic acid assay^23^, with absorbance measured at 510 nm. The McCord and Fridovich method was used to assess the superoxide anion radical scavenging activity^24^ via a mixture of phosphate buffer (pH 7.8), NBT, EDTA, riboflavin, and NaCN. Absorbance was measured at 530 nm.

TBA-reactive substances (TBARS) were assessed in a rat liver homogenate (10%) to measure the extent of lipid peroxidation^25^. One milliliter of the clear liver homogenate was mixed with serial dilutions (3.1–100 mg/mL, twofold dilution) of BVVO, UnSap, or Asc. Lipid peroxidation was then started by adding 100 µL of a 15 mM FeSO_4_ solution. Finally, a complex of lipid peroxidation products and TBA (0.67%) was formed and measured at 535 nm.

Ferric reducing power was assessed via the potassium ferricyanide–ferric chloride method^26^. BVVO, UnSap, or Asc was added at various concentrations (3.1–100 mg/mL, twofold dilution) separately to 0.2 M phosphate buffer (pH 6.6), 1% potassium ferricyanide, and 1% ferric chloride. Finally, absorbance was measured at 700 nm.

### Evaluation of the anticancer influence of UnSap on HepG-2 cells

#### Cytotoxicity assay

The MTT assay was used to determine the cytotoxicity of the extracts in normal and cancer cells. Peripheral blood mononuclear cells (PBMCs) are the normal cell type used to determine the safe doses of the studied extracts.

PBMCs were isolated from heparinized human blood following the Egyptian Ministry of Health and Population and National Health and Medical Research Council guidelines^96^. The present experiment was approved by the Human Research Ethical Committee (REC) of the Faculty of Medicine, Alexandria University, Egypt (approval number 0305386). After Ficoll-Hypaque density gradient centrifugation at 2000 rpm for 30 min, the PBMCs were isolated, resuspended in RPMI 1640 containing FBS (10%), and stained with 0.5% trypan blue to determine cell viability. Following the Mosmann method^28^, the cytotoxicity assay was performed by seeding 1 × 10^5^ PBMCs/well in a 96-well cell culture plate. The cells were then incubated with different concentrations (1, 0.5, 0.25, 0.125, and 0.0625%) of BVVO, UnSap, or 5-FU in DMSO (0.1% “v/v”) for 3 days (72 h) in a 5% CO_2_ incubator. Control cells were processed identically to the treated cells, receiving complete medium with the same DMSO concentration but without samples. Afterwards, 20 µL of MTT solution (5 mg/mL) was added to each well and incubated for 4 h at 37 °C. After the removal of the MTT solution, 150 µL of DMSO was added, and the absorbance of each well was recorded at 570 nm via a microplate reader (BMG LabTech, Germany). The safe dose (EC_100_) and half-maximal inhibitory concentration (IC_50_) values of each studied extract and 5-FU were determined using GraphPad InStat software.

The anticancer activities of BVVO and UnSap were assessed using the HepG-2 cell line. Cells were cultured in RPMI 1640 medium supplemented with 10% FBS. The anticancer activity of the tested extracts was examined via the MTT assay^28^. Cancer cell suspensions (3 × 10^3^ cells/well) were seeded into sterile 96-well flat-bottomed cell culture plates and allowed to attach for 24 h. Then, serial concentrations of BVVO, UnSap, or 5-FU were added to the cancer cells, which were subsequently incubated in a 5% CO_2_ incubator at 37 °C for 72 h. After incubation, MTT solution was added to each well, and the procedure described above was performed. The anticancer activities of BVVO and UnSap, compared with those of 5-FU, were assessed by calculating the IC_50_ values via GraphPad InStat software. In addition, the selectivity index (SI) of BVVO and UnSap versus 5-FU was calculated by dividing the IC_50_ value in normal PBMCs by the IC_50_ value in HepG-2 cells.

#### Assessment of caspase 3/7 activity

HepG-2 cells were incubated with BVVO, UnSap, or 5-FU (at their IC_50_ values) for 72 h in a CO₂ incubator. Following the manufacturer’s instructions, 100 μL of Caspase-Glo 3/7 substrate was mixed with the cells and incubated for 2 h. Afterwards, the plates were analyzed via a fluorescence Omega microplate reader (BMG LabTech, Germany) at 490 nm excitation and 520 nm emission.

### Real-time quantitative polymerase chain reaction (qRT‒PCR) of the cell cycle gatekeeper genes

Total RNA was extracted from treated and untreated HepG-2 cells via the GeneJET RNA Purification Kit, and cDNA was subsequently synthesized via the cDNA Synthesis Kit. The target genes and their primers used for SYBR Green qRT‒PCR are listed in Table 1. The qRT‒PCR program proceeded as follows: 15 min of enzyme activation (one cycle) at 95°C, followed by 15 s of denaturation (40 cycles) at 95°C, 1 min of annealing at 60°C, and 30 s of extension at 72°C. The fold change in the expression of these genes was determined before and after treatment with BVVO, UnSap, or 5-FU via the 2^-ΔΔCT^ equation. All gene expression levels were normalized to that of glyceraldehyde-3-phosphate dehydrogenase (*GAPDH*), a housekeeping/reference gene.

**Table 1:** Primer sequences of the selected genes of human and mice for qRT-PCR.

Forward primer (5´ 3')	Reverse primer (5′ 3')	Primer name	Function
Human primers			
CAAACTGGTGCTCAAGGCCC	GGGCGTCCCAAAGTAGGAGA	BAX	Apoptosis
GCGTGTTTGTGCCTGTCCTG	TGGTTTCTTCTTTGGCTGGG	TP53	
CTGGTGGACAACATCGCCCT	TCTTCAGAGACAGCCAGGAGAAAT	BCL2	
GACCCAGAAGCCATTGAAATCT	GGTGTGCTGGAAAAGGGTCC	RB1	Cell cycle regulator
TACCCTTGTGCCTCGCTCAG	GGCGGATTAGGGCTTCCTCT	CDKN1A	
GCATCCAGTGGAAGGGTGTG	ACGTTCCGGATGCTCTGCT	E2F4	
ACTGAATATAAACTTGTGGTAGTTGGACCT	CAAATCACATTTATTTCCTACCAGGACCAT	KRAS	Cell proliferation
TCAAGATCTGCCGAC TGAAC	CCTCTTTCTGCACCTTGTCA	NFKB1	Pro-inflammatory mediator
AGAAGGCTGGGGCTCATTTG	AGGGGCCATCCACAGTCTTC	GAPDH	Housekeeping gene
Mouse primers			
TAACAGTTCCTGCATGGGCGGC	AGGACAGGCACAAACACGCACC	TP53	Apoptosis
AGACAGGGGCCTTTTTGCTAC	AATTCGCCGGAGACACTCG	BAX	
CCCTATTTCATCTGCGACGAG	GAGAAGGACGTAGCGACCG	MYC	
GAGAGCGTCAACAGGGAGATG	CCAGCCTCCGTTATCCTGGA	BCL2	
AGAAGAGACGATGGACTTCCG	TCAAACTCGTTCATGGTCACAC	AKT1	
CTCAAACTGCCCAGCTTAACCC	TGCGGCTGACTGTGTAAGCAGA	GLI1	Cell cycle regulator
CCCTTGCTCTGCCTAACGC	GGAGTCCTGGCATCGTTGG	NOTCH1	
GATGACGGCGACATGGTTTAC	CTCACTGGGCCATTTCTGTGT	HIF1A	Angiogenesis marker
ACTGGGGCTGTGTGGAAAG	GCATTGAAGGTATCTTGGGTCTC	PROM1	Cancer stem cell marker
AACACAGGTTGGCAAGTTAATCA	TGCGACACAACATTGGCCTT	ALDH1A1	
TCAAGATCTGCCGAC TGAAC	CCTCTTTCTGCACCTTGTCA	NFKB1	Pro-inflammatory mediator
TGCACTATGGTTACAAAAGCTGG	TCAGGAAGCTCCTTATTTCCCTT	PTGS2	
CTGAACTGCCCTACCTACCAC	GAAGATGCCCAGGACGAT	ABCG1	Anti-inflammatory mediator
TGGCCTTCCGTGTTCCTAC	GAGTTGCTGTTGAAGTCGCA	GAPDH	Housekeeping gene

BAX, BCL-2-associated X-protein; TP53, tumor protein p53; BCL2, B-cell lymphoma; RB1, retinoblastoma 1; CDKN1A, cyclin-dependent kinase inhibitor; E2F4, E2 promoter binding factor 4; KRAS, Kirsten rat sarcoma virus; NFKB1, nuclear factor-KAPPA B; GAPDH, Glyceraldehyde 3-phosphate dehydrogenase; MYC, myelocytomatosis oncogene; AKT1, protein kinase B; GLI1, Glioma-associated oncogene; NOTCH1, neurogenic locus notch homolog protein; HIF1A, hypoxia-inducible factor; PROM1, prominin-1; ALDH1A1, aldehyde dehydrogenase 1A1; PTGS2, prostaglandin-endoperoxide synthase 2; and ABCG1, ATP-binding cassette subfamily G member.

### Heatmap plot and QIAGEN Ingenuity Pathway Analysis (IPA) for the studied genes

In the present study, a hierarchical clustering heatmap was used to illustrate the expression of the tested genes to identify the anticancer therapeutic potency of BVVO and UnSap and to compare their influence with that of 5-FU. A heatmap plot was created using the ClustVis web tool (https://biit.cs.ut.ee/clustvis/) to display multivariate data clustering^29^.

Network analysis was performed via IPA tool v.9 (Ingenuity Systems, Redwood, CA, USA; www.ingenuity.com) to cluster genes based on biological function. All investigated genes were obtained from Ensembl (https://www.ensembl.org/index.html), and both upregulated and downregulated genes were evaluated simultaneously to analyze possible functional correlations.

### In silico* studies*

#### Three-dimensional (3D) structural models of proteins and phytochemicals

The crystal structures of β-catenin and HTCF-4 (Protein Data Bank “PDB”: 1JDH), EGFR (PDB: 4WKQ), NOX2 (PDB: 3A1F), and SMO (PDB: 4JKV) were obtained from the PDB website (PDB, https://www.rcsb.org/). These proteins are important regulators of HCC proliferation and stemness. Additionally, the 3D structures of the most abundant UnSap constituents (rutin "RU, CID_5280805," benzoic acid "BA, CID_243," β-sitosterol "CID_222284," and stigmasterol “CID_5280794”) were gained from the PubChem database (https://pubchem.ncbi.nlm.nih.gov/) and used in the analysis.

#### Molecular docking analyses

The protein crystal structure was pre-processed for docking analysis by removing all crystallographic water molecules and co-crystallized small molecules and adding polar hydrogens to allow precise ligand–protein interaction analysis. The 3D structure of each studied UnSap constituent was docked with β-catenin, EGFR, NOX2, or SMO using the HDOCK web server (http://hdock.phys.hust.edu.cn/)^30^. The top-ranked docked complexes were examined and visualized with the Discovery Studio 2020 Client (v20.1.0.19295). Protein–ligand interaction interfaces were investigated, and 2D interaction diagrams demonstrating the binding of UnSap phytochemicals to target proteins were created using the same software.

#### Analysis of the docked complexes’ interface

The binding affinity, indicated by the change in Gibbs free energy (Δ^i^G) upon interface formation, was analyzed for the docked complexes using the Proteins, Interfaces, Structures, and Assemblies (PDBePISA) web server^31^. The corresponding interface surface area (Å^2^) was also measured.

The active site residues of the target proteins (EGFR, NOX2, and SMO) were retrieved from the PDBsum database (http://www.ebi.ac.uk/pdbsum) using their PDB IDs^32^. The expected competitive inhibitory potential of the UnSap constituents was determined by comparing the binding site residues identified in each docked complex with the corresponding reported active site residues. In addition, the binding site residues in each β-catenin-phytochemical docked complex were compared with the HTCF-4 binding residues of β-catenin.

### Evaluation of the anti-liver cancer effect of UnSap in mice

#### Animals and experimental design

Forty-two female albino mice (weight: 20–25 g; age: 4–6 weeks) were purchased from the Faculty of Medicine, Alexandria University, Alexandria, Egypt. The mice were housed in plastic cages for 15 days at 25°C, 55–65% relative humidity, and a natural day‒night cycle, and the animals were fed a conventional laboratory diet and given water. All animal experiments were conducted in accordance with the ethical criteria outlined in the Public Health Guide for the Care and Use of Laboratory Animals (National Research Council, 1996). All methods were performed in accordance with the Institutional Animal Care and Use Committee (IACUC) at Alexandria University (AU/04/19/03/23/03), granted on March 19, 2023. This study was reported in accordance with the Animal Research: Reporting of In Vivo Experiments (ARRIVE) guidelines (https://arriveguidelines.org).

The mice were randomly allocated to six groups (Fig. 10A), with ten mice per group. The groups were as follows: negative control group (C group), HCC group (HCC-bearing mice), HCC– UnSap group (HCC-bearing mice treated for 6 days with UnSap, 150 mg/kg^33^), HCC–5-FU group (HCC-bearing mice treated for 6 days with 5-FU, 20 mg/kg^19^), UnSap group (healthy mice administered UnSap only), and 5-FU group (healthy mice administered 5-FU only). The protocol described by *.* Shaban et al*.*^33^ was used to induce HCC in mice via *p*-DAB and PB. HCC induction was confirmed on day 61 by histopathological results prior to treatment with UnSap or 5-FU.

During the study, some animals died due to tumor-related mortality and other model-related issues. To ensure consistency across all groups, the final analysis was conducted using n = 7 animals per group, which represented the smallest number of surviving animals shared by all groups. This method enabled a balanced comparison of outcomes while maintaining the ethical ideals of reducing unnecessary animal use.

#### Blood and liver collection

At the end of the experimental period, the mice were anesthetized with isoflurane (2–3%) administered via a calibrated inhalation chamber until loss of consciousness was verified by the lack of reflex reactions (e.g., pedal withdrawal and corneal reflex). Once deep anesthesia was attained, the mice were euthanized by cervical dislocation. Blood was collected from the posterior vena cava of euthanized mice for serum preparation and stored at − 20°C for further examination. The liver tissues were subsequently removed from all groups of mice, cleaned with 0.9% NaCl, and cut into small pieces. For histopathological examination, small pieces were preserved in 10% formalin solution, and the remaining tissue portions were stored at − 80°C for subsequent biochemical and molecular analyses.

#### Serum analysis

The activities of AST, ALT, ALP, and γ-GGT were determined using specific kits. Additionally, total protein and albumin levels in the sera of all groups of mice were investigated.

#### Assessment of TBARS and GSH levels

The levels of lipid peroxidation (TBARS) and GSH in the clear liver homogenate (liver tissue was homogenized in PBS “1:10 wt/v”). The TBARS level was assessed colorimetrically at 532 nm, as described above via the TMP calibration curve^25^. Similarly, the GSH (non-enzymatic antioxidant) level was determined colorimetrically via Ellman’s reagent (5,5′-dithio bis2-nitrobenzoic acid), and a GSH calibration curve was generated^35^.

#### Histopathological study

Following the typical histopathological examination methodology, formalin-fixed tissue samples were embedded in paraffin wax, cut into tiny slices (5 µm thickness), and stained with hematoxylin and eosin. A phase-contrast microscope was subsequently used to visualize the abnormal characteristics of the analyzed tissues in all of the groups studied, and high-resolution photographs (20 × magnification) were taken^18^.

### Molecular analysis

The effects of UnSap administration to HCC-bearing mice for 6 days on the expression of thirteen target genes (antiapoptotic, apoptotic, metastatic, stem cell, and proliferation markers) were assessed using specific primers (Table 1). The isolated liver tissues were homogenized in lysis buffer containing β-mercaptoethanol and centrifuged for 5 min to obtain clear supernatants at 14,000 rpm. The total RNA in the prepared supernatants was subsequently extracted, and the procedure was completed as described above. All gene expression levels were normalized to *GAPDH* as a housekeeping/reference gene.

### Statistical analysis

The data of the current study are presented as the mean ± SE of three results (n = 3) for the in vitro antioxidant activity assays, five results (n = 5) for the in vitro anticancer activity assays, or seven mice for the in vivo study. One-way analysis of variance (ANOVA) was used with Duncan’s test to determine the difference between the mean values. The analysis was performed via the SPSS program v. 16, and significance was achieved at^*^p (^#^*p)* < 0.05,^**^p (^##^*p)* < 0.01, and^***^p (^###^*p)* < 0.0001. The GraphPad InStat program v. 3 was used to calculate the IC_50_ and EC_100_ values.

## Results

### Phenolic and phytosterol contents of UnSap

**Fig. 1:**
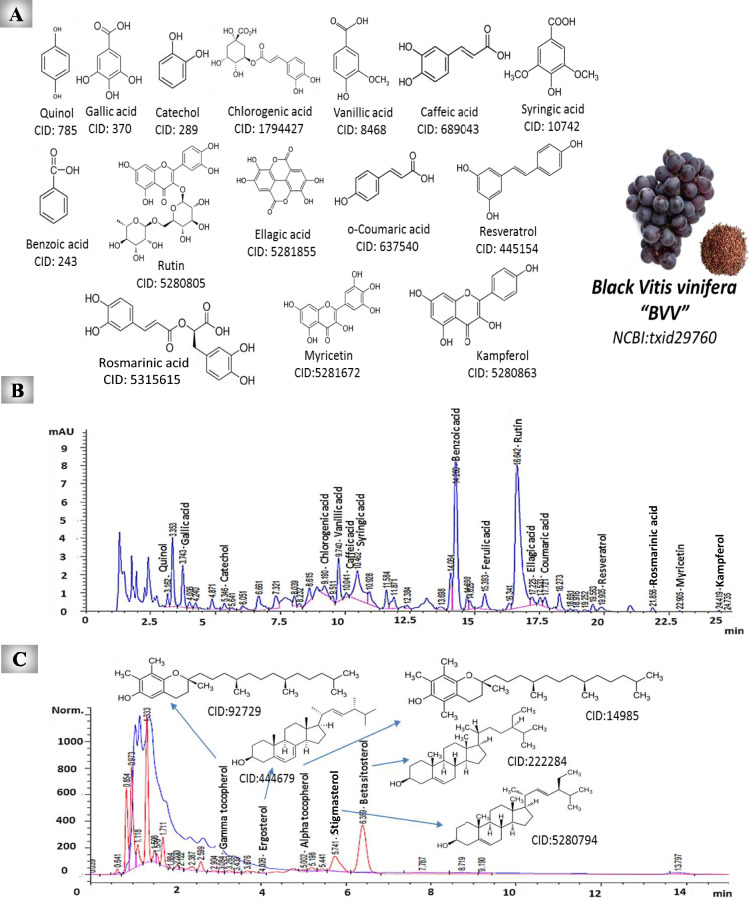
HPLC analysis of the (B) phenolic, (C) phytosterol, and tocopherol constituents of black* Vitis vinifera* seed oil (BVVO) unsaponifiable fraction (UnSap).** (A)** Structures of the phenolic and phytosterol constituents of UnSap extracted from the PubChem database (https://pubchem.ncbi.nlm.nih.gov/). The taxonomy ID of BVV was obtained from the NCBI database (https://www.ncbi.nlm.nih.gov/Taxonomy/Browser/wwwtax.cgi).

The present study evaluated the phenolic, phytosterol, and tocopherol constituents of UnSap via HPLC analysis. The results revealed that UnSap contains considerable amounts of these components (Fig. 1A- C). The phenolic compounds included (µg/g UnSap) quinol (0.547), gallic acid (0.850), catechol (0.689), chlorogenic acid (0.297), vanillic acid (0.792), caffeic acid (0.106), syringic acid (1.427), and BA (60.097). Additionally, ferulic acid (0.403), RU (33.527), ellagic acid (0.088), o-coumaric acid (0.502), resveratrol (1.367), rosmarinic acid (7.397), myricetin (6.110), and kampferol (1.941) are among the phenolic compounds in UnSap. However, pyrogallol, catechin, *p*-hydroxybenzoic acid, *p*-coumaric acid, cinnamic acid, quercetin, and naringenin were not detected in this extract.

HPLC analysis revealed that the phytosterols and tocopherols in UnSap (µg/g UnSap) included ergosterol (67.394), stigmasterol (8529.161), β-sitosterol (88,581.200), γ-tocopherol (29.329), and α-tocopherol (3207.320). However, UnSap contains no cholesterol.

### Antioxidant characterization of UnSap

The results revealed that the yields of BVVO (g/100 g of seeds) and UnSap (g/100 g of oil) were 2.2 ± 0.041 and 14.360 ± 0.665, respectively.

**Fig. 2:**
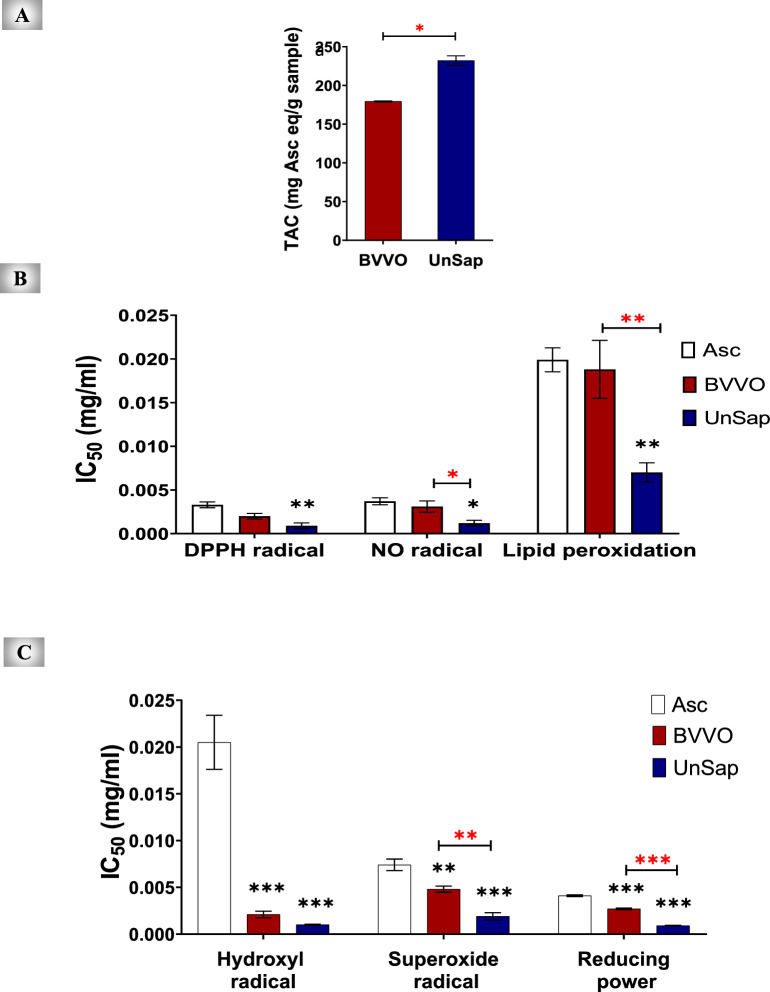
Antioxidant activities of black* Vitis vinifera* seed oil (BVVO) and its unsaponifiable fraction (UnSap) compared with ascorbic acid (Asc).** (A)** Total antioxidant capacity (TAC) of the studied extracts. **(B)** IC₅₀ values for the scavenging of DPPH, nitric oxide (NO), and lipid peroxide radicals.** (C)** IC₅₀ values for the scavenging of hydroxyl and superoxide radicals, along with the reducing power of the extracts. The results are expressed as the mean ± S.E. (n = 3). Statistical significance was considered at^*^p < 0.05,^**^p < 0.01, and^***^p < 0.0001. The black asterisks indicate comparisons with Asc, whereas the red asterisks represent comparisons between BVVO and UnSap. The ***IC***_***50***_ refers to the concentration of BVVO, UnSap or Asc that inhibits 50% of radical activity or achieves an absorbance of 0.5 in the reducing power assay.

As shown in Fig. 2A, the UnSap fraction exhibited a significantly (*p* < 0.05) greater TAC than BVVO. With respect to antiradical activities (Fig. 2B and 2C), UnSap demonstrated markedly stronger scavenging activity across all the tested radicals, as indicated by lower IC₅₀ values (*p* < 0.05, *p* < 0.01, and *p* < 0.0001) and greater reducing power than Asc, the reference antioxidant. Similarly, it had higher antioxidant efficiency than BVVO, except for DPPH and hydroxyl radicals, which showed the same potency.

### Anticancer efficiency of UnSap

**Fig. 3:**
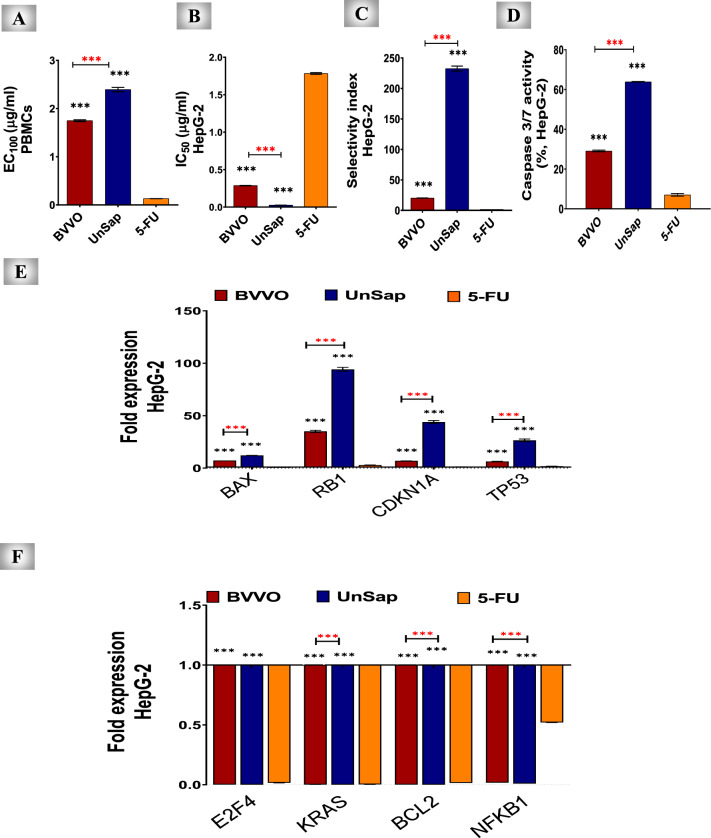
Apoptotic effects of black *Vitis vinifera* seed oil (BVVO) and its unsaponifiable fraction (UnSap), and their ameliorating effects on the expression of cell cycle-related genes in HepG-2 cells, compared with those of 5-FU. **(A)** EC_100_ values of BVVO and UnSap in peripheral blood mononuclear cells (PBMCs) compared with those of 5-FU. ***EC***_***100***_, the concentration of the studied sample that caused 100% viability of the PBMCs. **(B, C)** The IC_50_ and selectivity index (SI) values, respectively, of BVVO, UnSap, and 5-FU. The ***IC***_***50***_, the inhibitory concentration of BVVO, UnSap, or 5-FU, caused 50% inhibition of cancer cell growth. The SI is determined by dividing the cytotoxic concentration (the concentration of the sample that inhibits 50% of the growth of normal viable cells) by the IC_50_ value in cancer cells.** (D)** Percentage of caspase 3/7 activity in the HepG-2 cell line. **(E)** *BCL-2*-associated X-protein "*BAX*", retinoblastoma “*RB*” *1*, cyclin-dependent kinase inhibitor “*CDKN1A*”, and tumor protein “*TP53*” fold expression.** (F)** E2 promoter binding factor “*E2F*”*4*, Kirsten rat sarcoma virus “*KRAS*”, B-cell lymphoma “*BCL*”*2*, and nuclear factor “*NF*”*KB1* fold expression. The results are presented as the mean ± S.E. (n = 5), and significance was achieved at^*^p < 0.05,^**^p < 0.01, and^***^p < 0.0001. (*, black) indicates a comparison with the 5-FU; (*, red) refers to the comparison between the treatment with BVVO and UnSap.

The present study assessed the safety (EC_100_) of BVVO and UnSap compared with 5-FU in normal human PBMCs. The results (Fig. 3A) showed that UnSap is significantly (*p* < 0.0001) safer than BVVO and 5-FU.

The anticancer influence of UnSap was examined in the HepG-2 cell line, and its efficiency was compared in terms of the IC_50_ and SI values with those of 5-FU. The results in Fig. 3B and 3C show that UnSap had a significantly (*p* < 0.0001) lower IC_50_ and higher SI values than BVVO and 5-FU did.

### Anticancer mechanisms of UnSap in HepG-2 cells

#### Induction of apoptosis

After 72 h of incubation of HepG-2 cells with UnSap, the caspase 3/7 activity of these cells was significantly (*p* < 0.0001) higher than that of BVVO-treated cells by 2.195-fold and of 5-FU-treated cells by 9.077-fold (Fig. 3D).

#### Regulation of cell cycle gatekeeper genes

As shown in Fig. 3E, both BVVO and its UnSap fraction significantly (*p* < 0.0001) upregulated the expression of proapoptotic and tumor-suppressor genes—including *BAX*, *RB1*, *CDKN1A*, and *TP53*—in HepG-2 cells compared with 5-FU. Notably, UnSap demonstrated superior efficacy (*p* < 0.0001) in enhancing gene expression. In contrast, compared with 5-FU, both BVVO and UnSap significantly (*p* < 0.0001) downregulated the expression of oncogenic and antiapoptotic genes, including *E2F4*, *KRAS*, *BCL2*, and *NFKB1* (Fig. 3F). UnSap demonstrated comparable efficacy to BVVO in reducing *E2F4* expression but was significantly (*p* < 0.0001) more potent in suppressing *KRAS*, *BCL2*, and *NFKB1* expression.

#### The functional relationships between the studied genes and other genes in HepG-2 cells

**Fig. 4:**
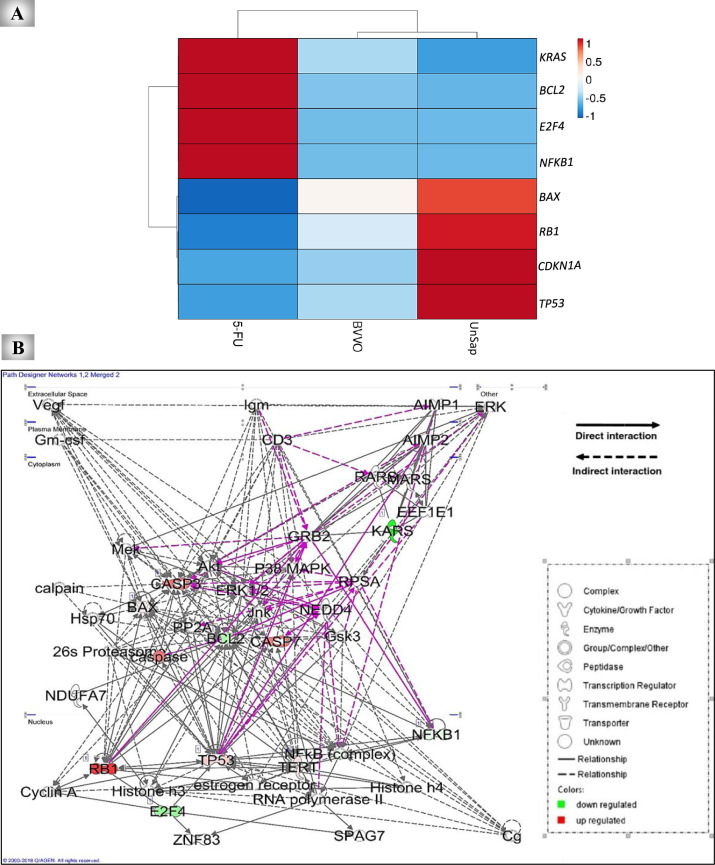
Heatmap plot and Ingenuity pathway analysis (IPA) network. **(A)** A heatmap showing the clustering of the expression of the investigated genes in HepG-2 cells. The color ranges from blue (low expression) to red (high expression). **(B)** QIAGEN IPA network of the proteins examined to evaluate the anticancer effects of the unsaponifiable fraction (UnSap) from black *Vitis vinifera* seed oil (BVVO) in comparison with 5-FU. *Green* denotes a significantly decreased level; *red* indicates a significantly increased level, according to color intensity. On the basis of the IPA database, molecular connections between interacting genes represent direct (solid line) or indirect (dotted line) functional links. The IPA legend describes the symbols representing the IPA networks provided in the inset.

A heatmap plot was generated to compare the anticancer effects of UnSap and 5-FU (Fig. 4A). This plot clustered the expression profiles of pro-apoptotic, anti-apoptotic, oncogenic, and tumor-suppressor genes in HepG-2 cells treated with UnSap, BVVO, and 5-FU. Gene expression levels are visually represented by color intensity, with red indicating upregulation and blue indicating downregulation. The heatmap clearly illustrates that UnSap exerted a more substantial proapoptotic effect than both BVVO and 5-FU did. Moreover, UnSap had a more pronounced anti-inflammatory effect and greater suppression of *KRAS*, *BCL2*, and *E2F4* expression than the other treatments did.

**Fig. 5:**
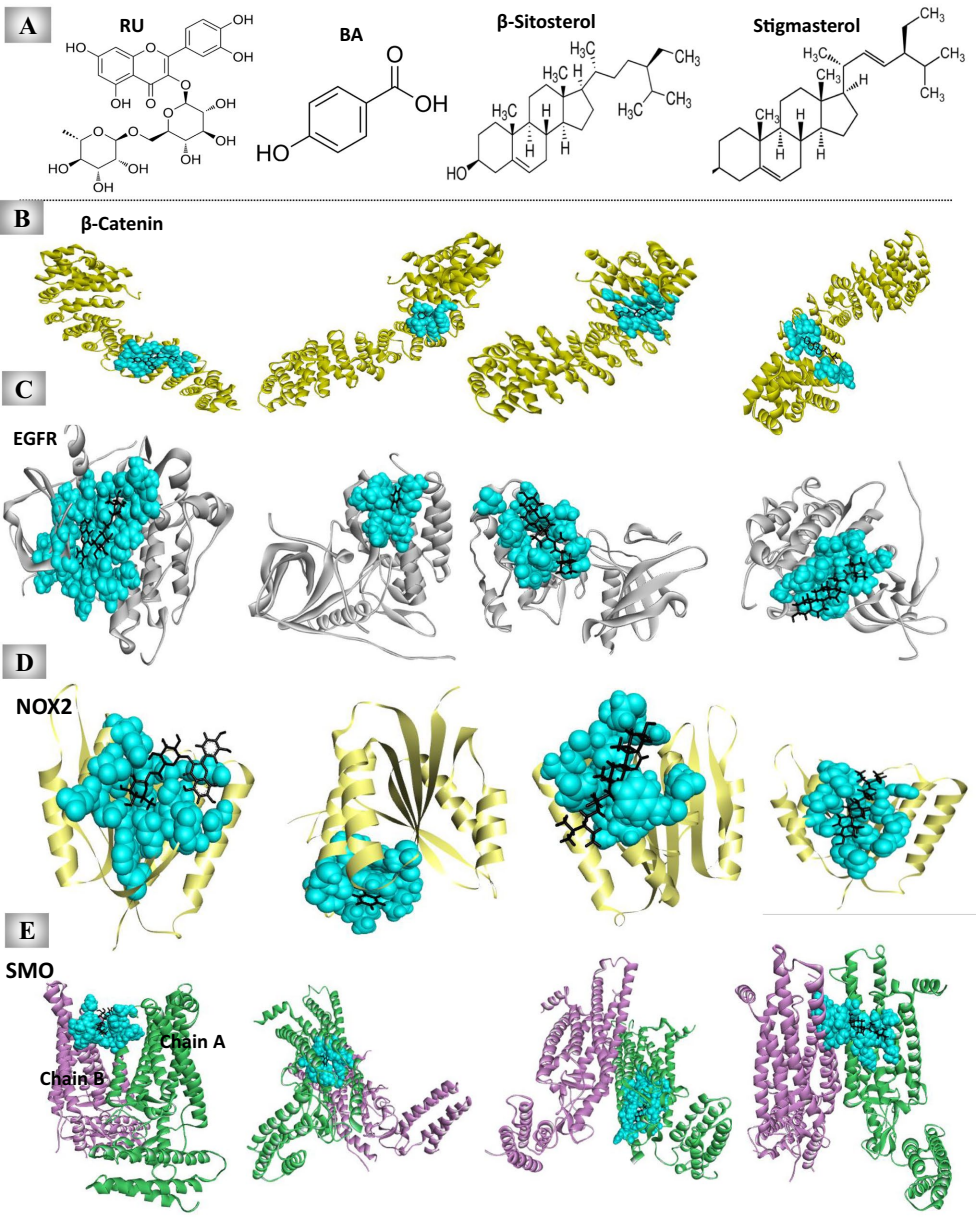
Molecular docking results of the most abundant constituents of the unsaponifiable fraction (UnSap) and the HCC key regulatory proteins. (**A**) The 2D structures of the most abundant constituents of UnSap, including rutin (RU), benzoic acid (BA), β-sitosterol, and stigmasterol. The structures were obtained from the PubChem database (https://pubchem.ncbi.nlm.nih.gov/). (**B-E**) The docked complexes between each of the UnSap constituents and β-catenin (Protein Data Bank “PDB”: 1JDH, shown in yellow cartoons), epidermal growth factor receptor kinase domain (EGFR, PDB: 4WKQ, shown in white cartoons), NADPH binding domain of NADPH oxidase 2 (NOX2, PDB: 3A1F, shown in pale yellow cartoons), and the smoothened receptor (SMO, PDB: 4JKV, shown in green “chain A” and purple “chain B” cartoons), respectively, were subsequently obtained by the HDOCK server (http://hdock.phys.hust.edu.cn/). The binding residues in the interface pocket are shown in cyan space-filling spheres. The Discovery Studio 2020 Client program (v20.1.0.19295) was used to analyze and visualize the generated docked complexes.

IPA revealed proteins related to cancer networks. The network revealed the protein‒protein interactions between the proteins under investigation and other vital proteins (Fig. 5B). Our proteins are linked to specific signaling pathways that are involved in cancer development or suppression, such as the mitogen-activated protein kinase (MAPK)/extracellular signal-regulated kinase (ERK) pathway, which plays important roles in cancer proliferation, angiogenesis, and metastasis. The Ras/MAPK/ERK signaling pathway is essential for cancer proliferation. Similarly, the c-Jun N-terminal kinase (JNK) protein can collaborate with NF-κB and p38 (MAPK) to promote cancer cell survival. The network identified the link between the 26S proteasome and TP53, BAX, and NF-κB. This protein has proteolytic action and helps in the degradation of cellular proteins, culminating in the activation of NF-κB and inhibition of TP53 and BAX. Other proteins, such as calpain, are detected by the IPA network as having cross-talk with caspase 3/7 and BAX. This protein, in conjunction with other apoptotic factors, plays an important role in the apoptosis-induced death of cancer cells.

#### Docking results

The current study chose the most abundant UnSap ingredients, based on HPLC results for phenolic and phytosterol quantity, for docking analysis, including RU, BA, β-sitosterol, and stigmasterol (Fig. 5A). Each of these components was docked with four target proteins: β-catenin (regulator of HCC stemness), EGFR (regulator of HCC proliferation), NOX2 (regulator of both HCC proliferation and stemness), and SMO (regulator of HCC stemness).

The molecular docking (Fig. 5B-E) revealed that the examined active constituents bound to the target proteins and were stable within their expected active or functional binding site. These findings lend evidence to the ability of the UnSap components to engage with molecular pathways related to HCC proliferation and stem cell maintenance.

#### The predicted blocking effect of UnSap constituents on β-catenin-HTCF-4 binding site

**Fig. 6:**
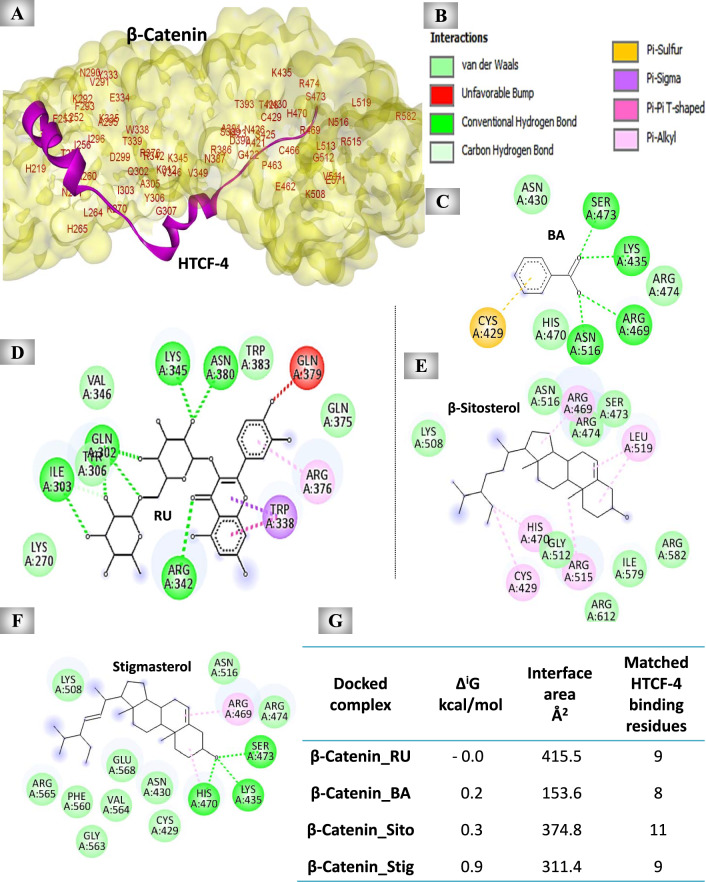
Molecular docking results of the most abundant constituents of the unsaponifiable fraction (UnSap) and β-catenin. (**A**) The docked complex of β-catenin (Protein Data Bank “PDB”: 1JDH, shown in yellow surface) and human T-cell factor-4 (HTCF-4, shown in purple cartoons). The binding residues in the interface pocket of the docked complex are shown in red. (**C-F**) The 2D structures of the binding residues in the interface pocket of the docked complexes of β-catenin and rutin (RU), benzoic acid (BA), β-sitosterol, or stigmasterol, respectively. (**B**) The molecular interactions observed in the docked complexes. (**G**) PDBe Protein Interfaces, Surfaces, and Assemblies (PISA) outcomes (Δ^i^G “binding affinity” and interface area) of the docked complex interfaces (https://www.ebi.ac.uk/pdbe/pisa/). The Discovery Studio 2020 Client software (v20.1.0.19295) was used to generate the 2D diagrams and to analyze the matching HTCF-4 interacting residues with the interface residues of each generated docked complex. The UnSap constituent with the highest binding affinity to the β-catenin is indicated by a red highlight.

The results showed that RU, BA, β-sitosterol, and stigmasterol individually bound to β-catenin by 16, 8, 13, and 14 amino acid residues, respectively. Among these compounds, Ru showed the highest binding affinity (the lowest Δ^i^G value) to β-catenin. Analysis of the interface pockets of β-catenin-HTCF-4 complex demonstrated that the HTCF-4 binding site encompasses 64 amino acid residues (Fig. 6A). Comparing these residues against the interface pockets of the docked complexes of RU, BA, β-sitosterol, or stigmasterol with β-catenin showed that these constituents can block the interaction site of HTCF-4 with different abilities (Fig. 6G). The 2D diagrams showed these binding residues, which interacted with β-catenin by different types of bonds (Fig. 6B-G).

#### The predicted inhibitory effect of UnSap constituents on the EGFR

**Fig. 7:**
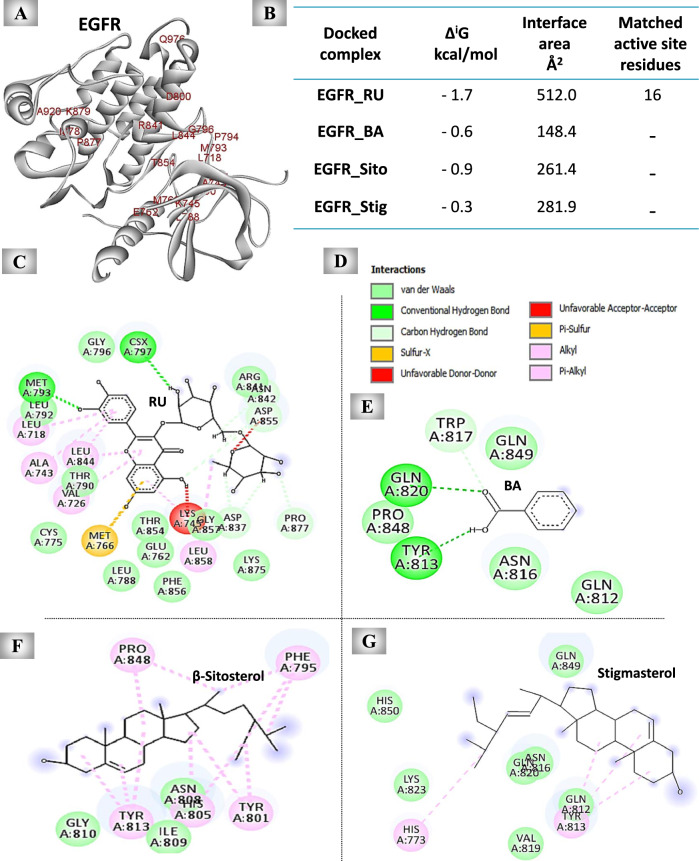
Molecular docking results of the most abundant constituents of the unsaponifiable fraction (UnSap) and epidermal growth factor receptor kinase domain (EGFR). (**A**) The crystal structure of EGFR (Protein Data Bank “PDB”: 4WKQ, shown in white cartoons). The active site residues of EGFR (retrieved from the PDBSum website “http://www.ebi.ac.uk/thornton-srv/databases/pdbsum/”) are shown in red. (**C, E–G**) The 2D structures of the binding residues in the interface pocket of the docked complexes of EGFR and rutin (RU), benzoic acid (BA), β-sitosterol, or stigmasterol, respectively. (**B**) PDBe Protein Interfaces, Surfaces, and Assemblies (PISA) outcomes (Δ^i^G “binding affinity” and interface area) of the docked complex interfaces (https://www.ebi.ac.uk/pdbe/pisa/). The Discovery Studio 2020 Client software (v20.1.0.19295) was used to generate the 2D diagrams and to analyze the matching active site residues of the EGFR with the interface residues of each generated docked complex. The UnSap constituent with the highest binding affinity to the EGFR is indicated by a red highlight. (**D**) The molecular interactions observed in the docked complexes.

As demonstrated in Fig. 7, RU, BA, β-sitosterol, and stigmasterol interacted with EGFR at 33, 10, 12, and 14 amino acid residues, respectively, showing different binding affinities, with RU having the highest affinity (Fig. 7B). EGFR has 20 active site residues (Fig. 7A), and only RU can interact with 16 of them, as shown in the 2D diagrams (Fig. 7C-G).

#### The predicted inhibitory effect of UnSap constituents on NOX2 activity

**Fig. 8:**
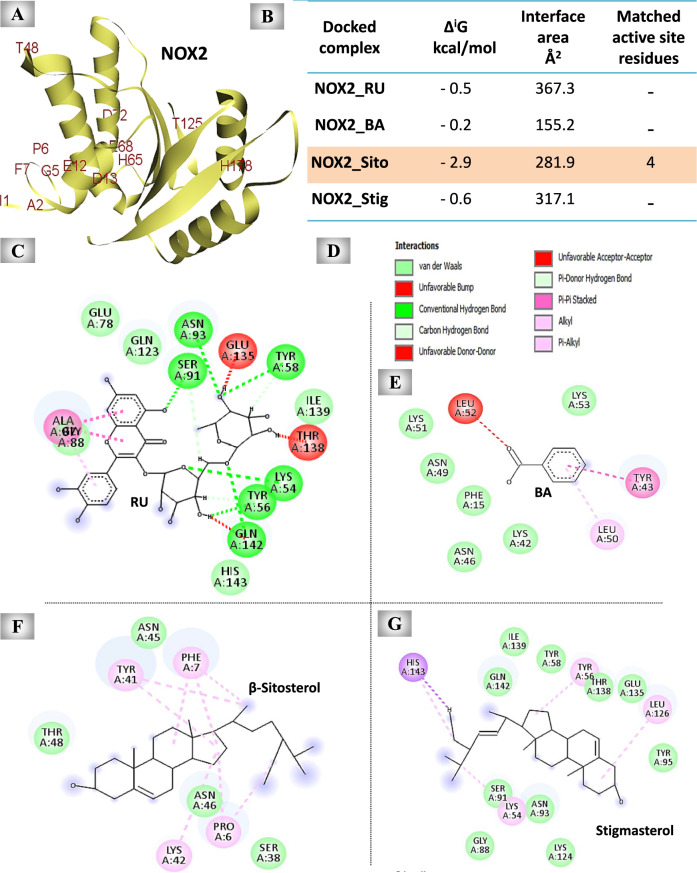
Molecular docking results of the most abundant constituents of the unsaponifiable fraction (UnSap) and NADPH binding domain of NADPH oxidase 2 (NOX2). (**A**) The crystal structure of NOX2 (Protein Data Bank “PDB”: 3A1F, shown in pale yellow cartoons). The active site residues of NOX2 (retrieved from the PDBSum website “http://www.ebi.ac.uk/thornton-srv/databases/pdbsum/”) are shown in red. (**C, E–G**) The 2D structures of the binding residues in the interface pocket of the docked complexes of NOX2 and rutin (RU), benzoic acid (BA), β-sitosterol, or stigmasterol, respectively. (**B**) PDBe Protein Interfaces, Surfaces, and Assemblies (PISA) outcomes (Δ^i^G “binding affinity” and interface area) of the docked complex interfaces (https://www.ebi.ac.uk/pdbe/pisa/). The Discovery Studio 2020 Client software (v20.1.0.19295) was used to generate the 2D diagrams and to analyze the matching active site residues of the NOX2 (retrieved from the PDBSum website “http://www.ebi.ac.uk/thornton-srv/databases/pdbsum/”) with the interface residues of each generated docked complex. The UnSap constituent with the highest binding affinity to the NOX2 is indicated by a red highlight. (**D**) The molecular interactions observed in the docked complexes.

The results showed that RU, BA, β-sitosterol, and stigmasterol interacted with different sites of NOX2 by 18, 12, 11, and 15 binding residues, respectively, with β-sitosterol demonstrating the strongest affinity (Fig. 8B). Furthermore, only β-sitosterol can bind with four amino acid residues of the enzyme active sites (NOX2 has 13 active site residues, Fig. 8A). The 2D diagrams revealed the binding residues of each examined molecule, as well as the types of interactions (Fig. 8C-G).

#### The predicted inhibitory effect of UnSap constituents on the SMO activity

**Fig. 9:**
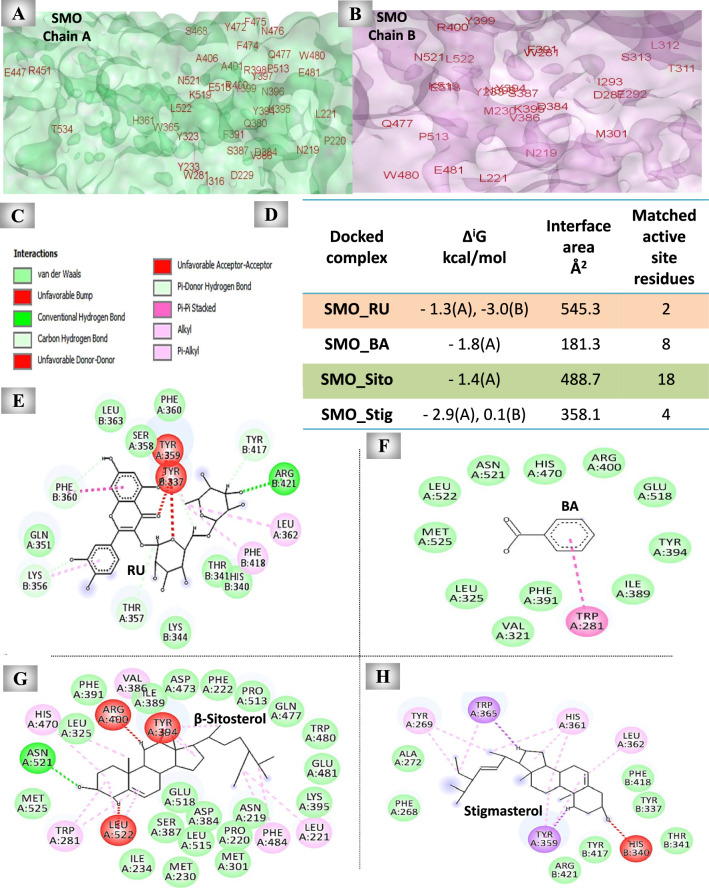
Molecular docking results of the most abundant constituents of the unsaponifiable fraction (UnSap) and smoothened receptor (SMO). (**A, B**) The crystal structure of SMO (Protein Data Bank “PDB”: 4JKV, shown in green “chain A” and purple “chain B” surface). The active site residues of the NOX2 (retrieved from the PDBSum website “http://www.ebi.ac.uk/thornton-srv/databases/pdbsum/”) are shown in red. (**E–H**) The 2D structures of the binding residues in the interface pocket of the docked complexes of SMO and rutin (RU), benzoic acid (BA), β-sitosterol, or stigmasterol, respectively. (**C**) The molecular interactions observed in the docked complexes. (**D**) PDBe Protein Interfaces, Surfaces, and Assemblies (PISA) outcomes (Δ^i^G “binding affinity” and interface area) of the docked complex interfaces (https://www.ebi.ac.uk/pdbe/pisa/). The Discovery Studio 2020 Client software (v20.1.0.19295) was used to generate the 2D diagrams and to analyze the matching active site residues of the SMO with the interface residues of each generated docked complex. The UnSap constituent with the highest binding affinity to the SMO is indicated by a red highlight, whereas that with the highest number of matched active site residues is indicated by a green highlight.

As shown in Fig. 9, molecular docking analysis revealed stable binding of UnSap constituents into the binding pocket of SMO, with different binding modes and interaction patterns (Fig. 9C, E–H). RU, BA, β-sitosterol, and stigmasterol interacted with SMO through 20 (7 on chain A and 13 on chain B), 16 (chain A), 35 (chain A), and 18 (12 on chain A and 6 on chain B) amino acid residues, respectively. PDBePISA analysis (Fig. 9D) showed that RU had the most favorable binding profile, with a greater binding affinity (the lowest Δ^i^G value) to both chains of SMO and a larger contact surface area than the other constituents. The results also showed that all the studied UnSap constituents can interact with certain active site residues of SMO, with β-sitosterol exhibiting the highest number of interactions (18 out of the 73 active site residues, Fig. 9A, B).

### Anti-HCC impacts of UnSap in HCC-bearing mice

#### Improvements of liver function parameters

This study evaluated the anti-HCC efficacy of UnSap in mice in comparison with that of 5-FU. After six days of treatment, UnSap significantly reduced the serum levels of ALP (*p* < 0.0001), γ-GGT (*p* < 0.0001), ALT (*p* < 0.0001), and AST (*p* < 0.0001) but significantly increased the total protein (*p* < 0.05) and albumin (*p* < 0.01) levels compared with those in the HCC group. In contrast, treatment with 5-FU over the same period resulted in a significant reduction in ALP (*p* < 0.0001) and an increase in AST (*p* < 0.01), without notable changes in the other liver function markers. Notably, UnSap demonstrated superior efficacy (*p* < 0.05, *p* < 0.0001) over 5-FU in improving ALP, γ-GGT, ALT, AST, total protein, and albumin levels by 1.541-, 1.727-, 1.300-, 1.171-, 1.228-, and 1.216-fold, respectively.

**Fig. 10:**
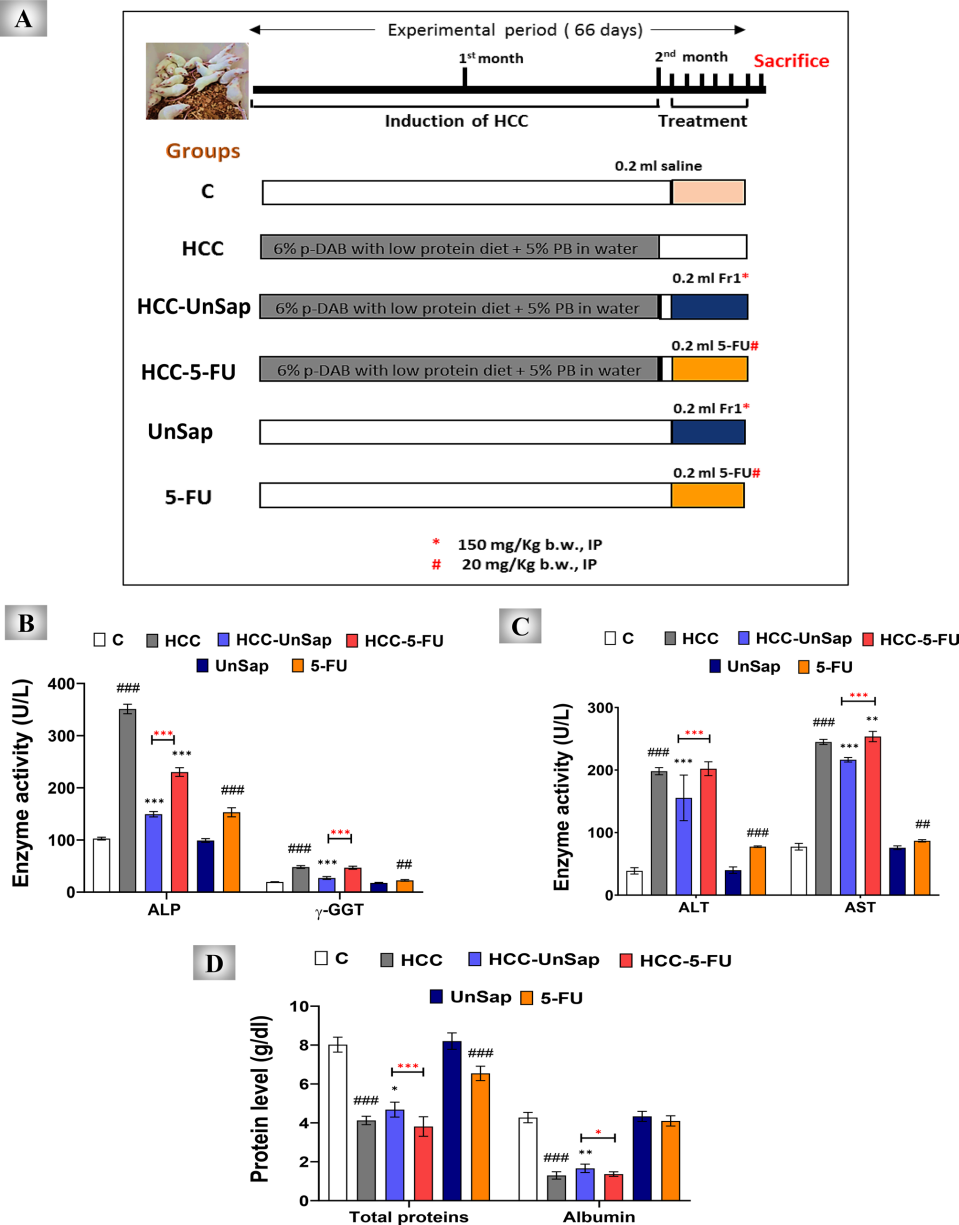
Experimental design of the therapeutic influences of an unsaponifiable fraction (UnSap) from *Vitis vinifera* seed oil on *p*-dimethylaminoazobenzene (*p*-DAB)-induced HCC in mice and its effects on serum liver function parameters. (**A**) The six different study groups. ***C***, control; ***HCC***, HCC-bearing mice; ***HCC-UnSap***, *Vitis vinifera* UnSap-treated mice. ***HCC-5-FU***, mice treated with 5-fluorouracil (FU); ***UnSap***, normal mice injected intraperitoneally (IP) for six days with UnSap; and ***5-FU***, normal mice injected (IP) for six days with 5-FU. The studied liver function parameters in the sera of the mice from all the studied groups included (**B**) ALP “alkaline phosphatase” and γ-GGT "γ-glutamyl transferase" activities. (**C**) ALT “alanine aminotransferase” and AST “aspartate aminotransferase” activities and (**D**) total protein and albumin levels. The results are presented as a mean ± S.E. (n = 7), and significance was achieved at^*^p (^#^*p)* < 0.05,^**^p (^##^*p)* < 0.01, and^***^p (^###^*p)* < 0.0001. (*, black) indicates a comparison with the HCC group; (*, red) refers to the comparison between HCC–UnSap and HCC–5-FU, whereas (#) represents a comparison with the control group.

Furthermore, UnSap administration to normal control mice did not significantly alter these parameters compared with those of the control group. Conversely, 5-FU treatment of healthy animals led to considerable increases in ALP (*p* < 0.0001), γ-GGT (*p* < 0.01), ALT (*p* < 0.0001), and AST (*p* < 0.01) levels and a marked reduction in total protein levels (*p* < 0.0001) relative to those in the control (Fig. 10B–D).

#### Amelioration of the hepatic redox state

**Fig. 11:**
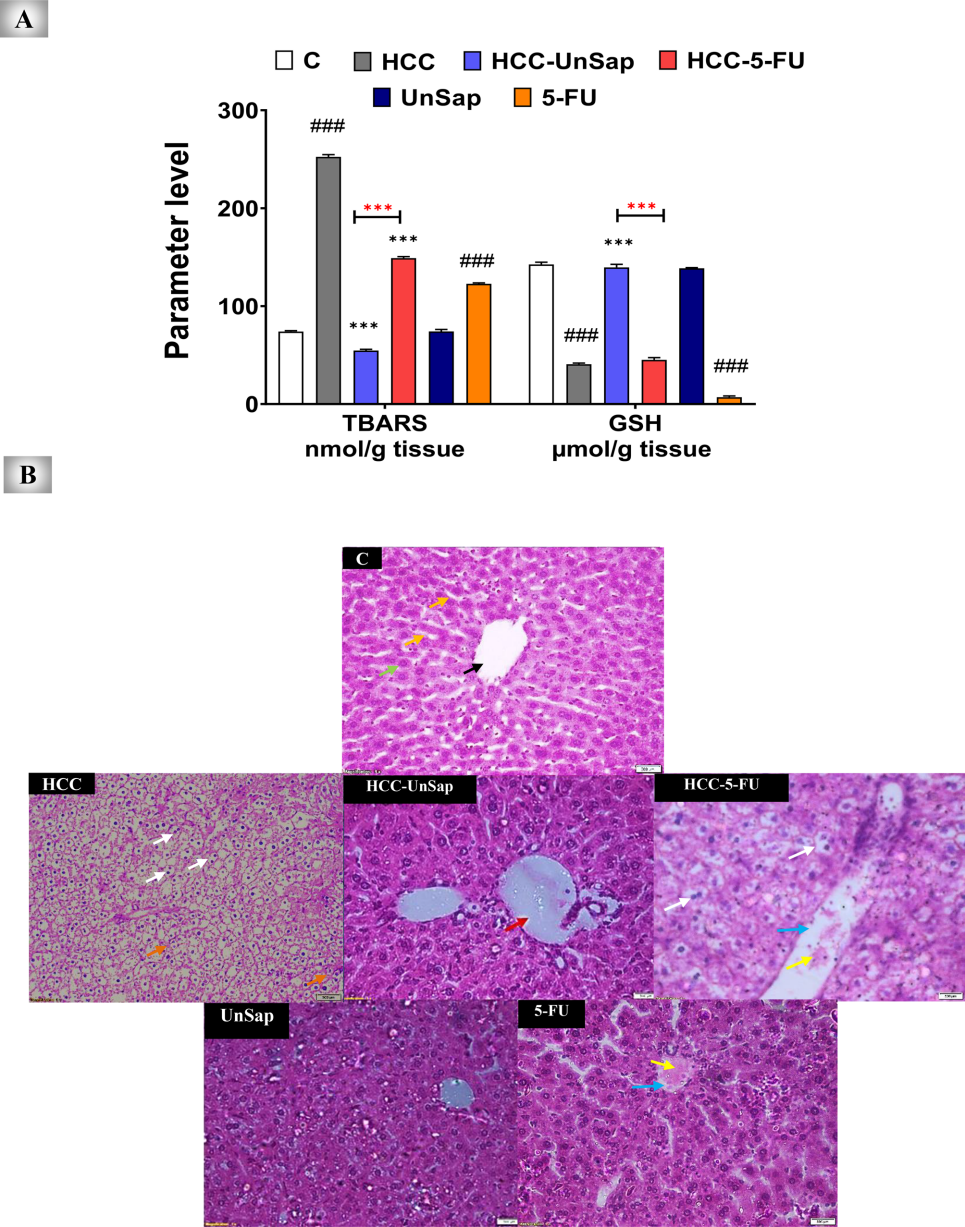
The attenuating effect of the unsaponifiable fraction (UnSap) from *Vitis vinifera* seed oil on *p*-dimethylaminoazobenzene (*p*-DAB)-induced hepatic cancer changes, lipid peroxidation, and depletion of GSH. (**A**) Thiobarbituric acid reactive substances (TBARS, lipid peroxidation) and GSH levels. The studied groups were as follows: ***C***, control; ***HCC***, HCC-bearing mice; and ***HCC-UnSap***, mice treated with *Vitis vinifera* UnSap. ***HCC-5-FU***, mice treated with 5-fluorouracil (FU); ***UnSap***, normal mice injected intraperitoneally (IP) for six days with UnSap; and ***5-FU***, normal mice injected (IP) for six days with 5-FU. The results are presented as a mean ± S.E. (n = 7), and significance was achieved at^*^p (^#^*p)* < 0.05,^**^p (^##^*p)* < 0.01, and^***^p (^###^*p)* < 0.0001. (*, black) indicates a comparison with the HCC group; (*, red) refers to the comparison between HCC–UnSap and HCC–5-FU, whereas (#) represents a comparison with the control group. (**B**) Hepatic morphology of the mice from all the examined groups. The arrows represent the central vein (black), hepatocytes (green), sinusoids (orange), tumor cells (white), fibrous bands (brown), dilated portal vein (red), blood vessel congestion and dilation (blue), and inflammatory cell infiltration (yellow).

Compared with the HCC group, six days of UnSap treatment significantly (*p* < 0.0001) reduced TBARS and increased GSH levels in the liver tissues of HCC-bearing mice (Fig. 11A). While 5-FU reduced TBARS levels (*p* < 0.0001) and did not affect GSH levels. Notably, UnSap exhibited greater efficacy than did 5-FU, improving TBARS and GSH levels by 2.724 - and 3.089 -fold, respectively.

Moreover, UnSap administration in healthy mice had no significant effect on these oxidative stress markers compared with those in normal control mice. In contrast, 5-FU treatment of normal mice significantly increased TBARS (*p* < 0.0001) and decreased GSH levels (*p* < 0.0001), indicating potential oxidative toxicity.

#### Improvement of hepatic architecture

Histological examination of liver tissues from healthy control mice revealed normal hepatic architecture characterized by intact central veins and well-organized hepatocytes within the parenchyma (Fig. 11B). In contrast, the liver tissues of the HCC-bearing mice presented marked pathological alterations, including tumor cell infiltration, dilated portal veins, inflammatory infiltrates, distorted sinusoids, and fibrous band formation. Treatment with UnSap in the HCC–UnSap group markedly restored normal hepatic architecture, with only mild central vein congestion observed. Conversely, liver tissues from the HCC–5-FU group presented persistent abnormalities, such as tumor cells, dilated and congested central veins, deformed sinusoids, and limited fibrous band formation.

Notably, UnSap administration in healthy mice did not induce any histopathological alterations, whereas 5-FU treatment in healthy mice led to central vein congestion, inflammatory cell infiltration, and mild sinusoidal distortion.

#### Regulation of HCC-related genes

**Fig. 12:**
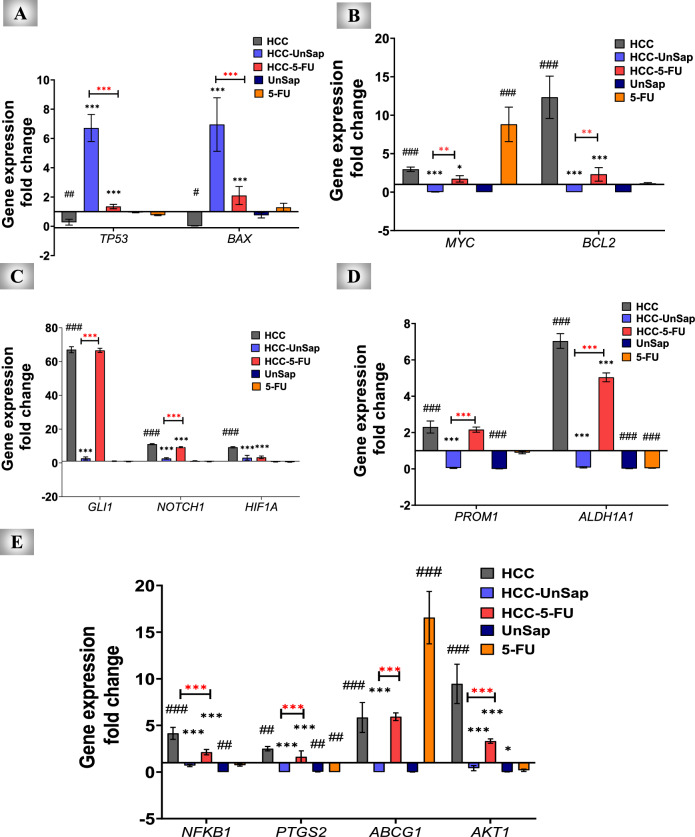
Ameliorating effect of the unsaponifiable fraction (UnSap) from *Vitis vinifera* seed oil on the relative expression of critical genes involved in HCC pathogenesis. (**A**) Tumor protein (*TP53*) and *BCL-2*-associated X-protein "*BAX*" fold changes in expression. (**B**) Myelocytomatosis oncogene "*MYC*" and B-cell lymphoma 2 "*BCL2*" fold expression. (**C**) Glioma-associated oncogene "*GLI1*", neurogenic locus notch homolog protein "*NOTCH*"*1*, and hypoxia-inducible factor "*HIF*"*1A*. (**D**) Prominin1 “*PROM1*” and the aldehyde dehydrogenase "*ALDH*"*1A1*. (**E**) Nuclear factor (*NF*)*KB1*, prostaglandin-endoperoxide synthase 2 “*PTGS2*”, ATP-binding cassette subfamily G member “*ABCG*” *1*, and protein kinase B (*AKT) onefold* expression. The studied groups were as follows: ***C***, control; ***HCC***, HCC-bearing mice; and ***HCC-UnSap***, mice treated with *Vitis vinifera* UnSap. ***HCC-5-FU***, mice treated with 5-fluorouracil (FU); ***UnSap***, normal mice injected intraperitoneally (IP) for six days with UnSap; and ***5-FU***, normal mice injected (IP) for six days with 5-FU. The results are presented as a mean ± S.E. (n = 7), and significance was achieved at^*^p (^#^*p)* < 0.05,^**^p (^##^*p)* < 0.01, and^***^p (^###^*p)* < 0.0001. (*, black) indicates a comparison with the HCC group; (*, red) refers to the comparison between HCC–UnSap and HCC–5-FU, whereas (#) represents a comparison with the control group.

As shown in Fig. 12A-E, the gene expression of *TP53* and *BAX* in the liver tissues of mice fed *p*-DAB and PB (HCC group) was significantly (*p* < 0.01, *p* < 0.05) downregulated. In contrast, all the other investigated genes were upregulated (*p* < 0.01, *p* < 0.0001). Compared with the HCC group, the treatment with UnSap (HCC–UnSap) showed significant (*p* < 0.0001) upregulation of *TP53* and *BAX* expression and significant (*p* < 0.0001) downregulation of all other tested genes. Similarly, treatment with 5-FU (HCC–5-FU group) led to a significant (*p* < 0.0001) increase in *TP53* and *BAX* expression. Conversely, it caused a substantial decrease in the expression of myelocytomatosis oncogene (*MYC*, *p* < 0.05), *BCL2* (*p* < 0.0001), neurogenic locus notch homolog protein (*NOTCH*)*1* (*p* < 0.0001), and hypoxia-inducible factor (*HIF*) *1A* (*p* < 0.0001). Similarly, the expression of aldehyde dehydrogenase (*ALDH*) *1A1* (*p* < 0.0001), *NFKB1* (*p* < 0.0001), prostaglandin-endoperoxide synthase 2 (*PTGS2*, which encodes cyclooxygenase “COX”-2) (*p* < 0.001), and protein kinase B (*AKT*) *1* (*p* < 0.01) was decreased. However, 5-FU did not affect the fold expression of glioma-associated oncogene (*GLI1*), prominin 1 (*PROM1*, which encodes CD133), or ATP-binding cassette subfamily G member (*ABCG*) *1*. The findings also demonstrated that UnSap was more effective than 5-FU in ameliorating the aforementioned genes in HCC-bearing mice.

On the other hand, the administration of UnSap for six days (UnSap group) to healthy animals significantly (*p* < 0.05, *p* < 0.01, *p* < 0.0001) reduced the fold changes in the expression of *PROM1*, *ALDH1A1*, *NFKB1*, *PTGS2*, and *AKT1* in liver tissues compared with those in the control group. However, injection of 5-FU for the same period (5-FU group) resulted in a significant (*p* < 0.0001) increase in the expression of *MYC* and *ABCG1* and a significant decrease in the expression of *ALDH1A1* (*p* < 0.0001) and *PTGS2* (*p* < 0.01). However, there were no significant changes in the fold changes of the expression of the other studied genes compared with the control group.

## Discussion

The current study evaluated the anticancer efficacy of UnSap in comparison with that of 5-FU. UnSap exhibited strong antioxidant activity, demonstrated greater cytotoxicity against HepG-2 cells than both 5-FU and BVVO, and had a safer effect on PBMCs. The cytotoxic effects of BVVO and UnSap may be due to their phytochemical constituents, including fatty acids (such as stearic, palmitic, oleic, and linoleic acids), phenolics, alkaloids, terpenoids, and others^36–38^. These components exhibit potent antioxidant activity, effectively scavenging free radicals and thereby preventing cellular damage and reducing the risk of cancer development^39^. Our recently published research revealed that the Sap matter of BVVO contains approximately twelve fatty acids (saturated and unsaturated), most of which are well known for their anticancer effects. These fatty acid constituents have potent apoptotic and anti-inflammatory effects on MCF-7 cells and Ehrlich ascites carcinoma by upregulating *TP53* and downregulating *BCL2* and *NFKB1*^19^. These results were in line with the previous studies of Erdogan et al*.* and Vishwasrao et al*.*^40–42^.

HPLC analysis revealed that the UnSap fraction of BVVO contains valuable phenolic compounds and phytosterols (Fig. 1). These phytochemicals exert proapoptotic effects on HepG-2 cells by activating caspase-3/7. Moreover, they modulate the expression of key cell cycle- and apoptosis-related genes, including *BAX*, *RB1*, *CDKN1A*, *TP53*, *E2F4*, *KRAS*, and *BCL2*. Modulation of these genes represents a promising therapeutic strategy to overcome cancer cell resistance to apoptosis. The interplay among these genes governs cell cycle progression and arrest. Among them, *TP53* (a major proapoptotic regulator), *BAX* (proapoptotic), and *BCL2* (antiapoptotic) serve as critical modulators of apoptotic signaling^43^. *TP53* is a tumor suppressor gene whose protein product regulates cell division and detects DNA damage to facilitate its repair. When DNA repair fails, TP53 promotes apoptosis by activating *BAX*, thereby initiating the intrinsic apoptotic pathway^44^. BAX translocates to the mitochondria, where it integrates into the outer membrane and forms pores, facilitating the release of proapoptotic factors and calcium ions. This mitochondrial permeabilization ultimately leads to the activation of caspases-3, -6, and -7. In contrast, BCL-2 is an antiapoptotic protein that inhibits apoptosis by preventing the release of calcium, thereby maintaining cellular homeostasis^43^.

RB1, a mitochondrial-associated tumor suppressor protein, is encoded by a gene that is mutated in approximately one-third of human cancers. RB1 can bind to BAX and promote BAX-dependent apoptosis^44^. In its active, dephosphorylated form, RB1 binds to the transcription factor E2F4 during the early G1 phase, thereby repressing its transcriptional activity and halting cell cycle progression. E2F transcription factors, particularly E2F4, are key regulators of the S, G2, and M phases of the cell cycle. Upon phosphorylation of RB1 by CDK4/6 in the mid-to-late G1 phase, E2F4 is released, allowing transcription of necessary G1/S transition genes and DNA synthesis. Both CDK4/6 and E2F4 play critical roles in promoting uncontrolled proliferation in cancer cells, including HepG-2 cells. Additionally, KRAS is an oncoprotein mutated in approximately 30% of cancers^45^. It plays a pivotal role in driving G1 CDK activation, RB1 phosphorylation, and S-phase entry, further contributing to tumorigenesis^46^.

NF-κB is a key proinflammatory mediator involved in regulating apoptosis, cell cycle progression, and the initiation and progression of malignancies, including HepG-2 cells^47,48^. As a transcription factor, NF-κB modulates the expression of various genes, including those encoding growth factors, cell adhesion molecules, cytokines, acute phase proteins, and apoptosis regulators^48,49^. Owing to its broad regulatory influence, constitutive activation of NF-κB has been strongly associated with various cancer types^19,47,52^. In this context, the ability of UnSap to upregulate the expression of tumor suppressor and cell cycle–regulating genes, such as *BAX*, *RB1*, *CDKN1A*, and *TP53*, highlights its promising role in cancer suppression. Conversely, UnSap downregulates the expression of oncogenic and antiapoptotic genes, including *E2F4*, *KRAS*, *BCL2*, and *NFKB1*. Regulating cell cycle progression through the modulation of checkpoint protein-encoding gene expression is a critical strategy for controlling carcinogenesis.

The anticancer potential of UnSap may be attributed to its high content of phenolic and phytosterol compounds. Previous studies have demonstrated that phenolics such as catechin, RU, kaempferol, and resveratrol can induce apoptosis in various cancer cell lines, including HepG-2 cells, by upregulating *TP53*, *BAX*, and caspases-3/7 while downregulating *BCL2* and *CDK1*^53,54^. Additionally, gallic, caffeic, ferulic, and *p*-coumaric acids, as well as myricetin, have shown potent anticancer and anti-inflammatory effects, often through the inhibition of the NF-κB pathway^54,55^. Resveratrol is also known to inhibit the NF-κB pathway and suppress angiogenesis in HCC^56^. Furthermore, ergosterol, stigmasterol, and particularly β-sitosterol—the most abundant phytosterol in UnSap—have exhibited both anti-inflammatory and pro-apoptotic properties in various cancer models^57,58^. Several studies have indicated that phytochemicals with antioxidant capabilities restore redox equilibrium, suppress NF-κB signaling, and induce TP53/BAX-dependent apoptosis in HCC models^59–61^. This may be owed to the fact that reduced oxidative stress can promote TP53 stability and functional activity, resulting in transcriptional upregulation of pro-apoptotic genes like *BAX* and subsequent induction of mitochondrial-mediated apoptosis^62,63^. Thus, UnSap’s antioxidant activity may provide an achievable molecular basis for the regulation of these genes and the subsequent antiproliferative and pro-apoptotic effects observed throughout this work. The results of the present study demonstrated that UnSap was more effective than 5-FU in modulating the expression of these key genes, as clustered in the heatmap plot.

The IPA network revealed functional associations between the previously discussed proteins and several critical signaling pathways, including the MAPK/ERK, JNK, 26S proteasome, and calpain signaling pathways. These pathways play pivotal roles in regulating cancer cell growth, survival, and progression. Inhibition of *KRAS* and *BCL2* by UnSap may lead to suppression of the MAPK/ERK pathway^46,53^, which is known to drive cancer cell proliferation, angiogenesis, and metastasis^45^. The JNK pathway, which is also mapped within the IPA network, is activated by cytochrome c and is a key mediator of the intrinsic apoptotic pathway^53^. The 26S proteasome, another protein highlighted in the network, is a recognized therapeutic target in cancer treatment. Its inhibition has been shown to increase the levels of proapoptotic proteins, such as TP53, BAX, and caspases, while reducing BCL*-*2 and CDK levels. However, proteasome activity can also paradoxically activate the NF-κB pathway, contributing to cancer progression. Thus, selective inhibition of the 26S proteasome promotes apoptosis and represents a promising anticancer strategy^64^. Notably, the phenolic and phytosterol constituents of UnSap may contribute to this inhibition. Prior studies have demonstrated that compounds such as ferulic acid and various flavonoids can effectively inhibit 26S proteasome activity^64,65^. Calpain, a calcium-activated protease also involved in the IPA network, is upregulated in numerous cancer types, including HepG-2 cells. It promotes cancer progression by activating AKT signaling, degrading tumor suppressors, such as *TP53* and *BAX*, and inactivating caspases-3/7, thereby impairing apoptotic processes^66^. Given its crucial role in maintaining cancer cell viability, calpain is considered an important therapeutic target. The regulatory impact of UnSap on these proteins needs further investigation to support its potential as an effective anticancer agent. Upregulation of the *NFKB1*/*KRAS* signaling axis and E2F, along with reduced expression of *RB1*, *TP53*, and *CDKN1A*, has been associated with increased cancer cell proliferation and maintenance of cancer stemness^47,67^. The antiproliferative and stem cell-targeting effects of UnSap are likely attributed to its bioactive composition, such as vanillic, caffeic, and syringic acids, as well as β-sitosterol and α-tocopherol^58,68^.

The docking results found that the most abundant constituents in UnSap, including RU, BA, β-sitosterol, and stigmasterol, target key signaling nodes involved in HCC progression. All four substances were expected to interfere with the β-catenin/HTCF-4 interaction by occupying key residues within the complex interface. This suggests a potential inhibition of Wnt/β-catenin-pathway and, thus, cancer stemness. Hence, aberrant activation of the Wnt/β-catenin signaling pathway is a critical factor in HCC development and is frequently detected in HCC patients. This pathway is critical for cancer stem cells’ stemness^69^. The interaction of β-catenin and HTCF-4 is critical for HCC cell proliferation and survival. Altering the β-catenin/HTCF-4 complex by UnSap ingredients decreases expression of downstream oncogenic targets and suppresses HCC cell proliferation^70^. In addition, UnSap constituents exhibited competitive inhibitory binding toward SMO. SMO is an important transducer of the Hedgehog (Hh) signaling pathway, and it is usually aberrantly active in HCC. Activation of SMO increases self-renewal, proliferation, epithelial-mesenchymal transition, and resistance to therapy in HCC cells, helping to maintain cancer stem cell features. Importantly, activating the Hh/SMO pathway has been demonstrated to upregulate CD133 expression, strengthening stemness features in HCC cancer stem cells^71^. Therefore, inhibition of this pathway by UnSap components may disrupt HCC stemness by attenuating CD133 expression and impairing self-renewal and tumor-initiating capacities of cancer stem cells. Notably, RU showed a strong competitive inhibition of the EGFR kinase domain. This result is in harmony with the previous study of Choi et al.^72^. EGFR is frequently overexpressed or activated abnormally in HCC, resulting in the activation of its intracellular tyrosine kinase domain and subsequent autophosphorylation. This activates a variety of oncogenic signaling pathways, which boost HCC cell proliferation, survival, invasion, and apoptosis resistance^73^. New evidence reveals that EGFR regulates HTCF-dependent β-catenin transcriptional activity in HCC via kinase-independent pathways^69^. Docking results also showed that β-sitosterol demonstrated competitive inhibition of the NOX2 activity. NOX2 promotes HCC development by increasing the production of reactive oxygen species (ROS), which causes oxidative stress, inflammation, and DNA damage associated with malignant transformation. Tumor-derived signals in HCC can activate NOX2 in immune cells like macrophages, resulting in macrophage polarization that promotes tumor growth^74^. Collectively, the multi-target inhibitory activities of the studied UnSap compounds highlight their efficacy to disrupt Wnt/β-catenin, Hh, EGFR, and NOX2 pathways, indicating their predicted anti-HCC effect.

The present study also investigated the impact of UnSap on *p*-DAB (HCC initiator)-induced HCC in mice through chronic feeding with PB (HCC promoter). Following metabolic activation, *p*-DAB undergoes N-dimethylation to form monoaminoazobenzene, which is subsequently converted into aminoazobenzene, a compound known to contribute to hepatic carcinogenesis^75^. The metabolic byproducts of *p*-DAB generate free radicals^76^, which attack cellular macromolecules, leading to structural and functional damage and the formation of lipid peroxides (TBARS)^75^. The accumulation of these free radicals disrupts the cellular redox balance and contributes to the development of HCC^77^. Consequently, elevated TBARS levels and decreased GSH levels were observed in the liver tissues of HCC-bearing mice. Moreover, chronic administration of *p*-DAB resulted in significant alterations in liver architecture and function. This was evidenced by increased serum activities of liver enzymes—ALT, AST, ALP, and γ-GGT—and a reduction in total protein and albumin levels. These findings reflect impaired hepatic function, as liver parenchymal cells are primarily responsible for serum protein synthesis, particularly that of albumin; thus, its decline serves as an indicator of liver injury^78^.

The accumulation of free radicals following *p*-DAB administration contributes to mutagenesis and tumor progression through activation of the NF-κB pathway and promotion of angiogenesis. In parallel, it upregulates antiapoptotic factors such as *BCL-2* while downregulating proapoptotic genes, including *BAX* and *TP53*^79^. Additionally, excessive free radical production and hypoxia are associated with *GLI1* overexpression in HCC-bearing mice. *GLI1*, a zinc finger transcription factor, inhibits apoptosis through *TP53* suppression and regulates the expression of *ABCG1*, a gene involved in cholesterol homeostasis^80^. The overexpression of *PTGS2* was also observed in HCC-bearing mice, contributing to the regulation of prostaglandin metabolism and promoting cancer metastasis, angiogenesis, and the activation of *AKT1* through phosphorylation^81^. *AKT1* functions as an oncogene and plays a key role in cell proliferation, survival, growth, and angiogenesis^81^. Similarly, *MYC*—another oncogene—is overexpressed in HCC and contributes to tumor proliferation by suppressing apoptosis^82^. Free radical accumulation can further induce *HIF1A*, a hypoxia-inducible factor that exacerbates hypoxic conditions and promotes additional *HIF1A* expression via NF-κB activation^83^. In the context of HCC, HIF*-*1α may activate the AKT pathway, thereby enhancing tumor metastasis and proliferation^83^. The current study also revealed that chronic *p*-DAB exposure led to a marked increase in the expression of *PROM1* to encode CD133, a cancer stem cell marker associated with resistance to chemotherapeutic agents such as 5-FU. CD133 activates NOTCH1, a transmembrane receptor that regulates cell proliferation and differentiation, and cooperates with HIF-1α to sustain cancer stem cell survival, contributing to poor prognosis^83^. Additionally, *ALDH1A1* and *ABCG1* were markedly overexpressed, both of which are associated with enhanced HCC proliferation and poor clinical outcomes. ABCG1 facilitates the efflux of excess cholesterol to high-density lipoproteins, whereas ALDH1A1 is a cytoplasmic enzyme essential for the synthesis of retinoic acid from retinaldehyde^84,85^. Collectively, these genes and their corresponding proteins represent critical therapeutic targets in HCC because of their integral roles in regulating cancer cell growth, survival, and progression.

UnSap treatment of HCC-bearing mice for six days showed a strong anti-HCC impact. UnSap was delivered via IP injection, a route that avoids gut first-pass metabolism but allows for partial hepatic first-pass metabolism through the portal circulation. This route is simple, quick, appropriate for repeated dosing, and causes the least amount of discomfort for rodents. The peritoneal cavity offers immediate access to systemic circulation after IP injection. Highly lipophilic substances are quickly absorbed systemically when given IP, according to earlier research. According to experimental data, fluids and small- to medium-sized molecules are mainly absorbed through the visceral peritoneum via the splenic, inferior, and superior mesenteric capillaries. They then enter the portal vein and, following partial hepatic metabolism, the systemic circulation^86^. Consistently, UnSap constituents likely may follow a similar absorption pattern, as their components have small molecular weights, as determined by our previous HPLC analysis. The current results showed that treatment of HCC-bearing mice with UnSap significantly reduced TBARS levels and elevated hepatic GSH concentrations. In addition, UnSap restored liver histological architecture, with only mild central vein congestion observed. Moreover, treatment improved the levels of serum liver function markers. These findings highlight the potent antioxidant activity of UnSap, which is likely attributed to its high content of phenolic compounds and phytosterols. Several phenolic acids and flavonoids present in UnSap, including resveratrol, ferulic, caffeic, gallic, ellagic, syringic, and cinnamic acids, as well as RU and kaempferol, are well documented for their antioxidant properties^55,87–89^. Phytosterols such as stigmasterol, ergosterol, and β-sitosterol also exhibit notable antioxidant effects^90^. Moreover, the docking results predicted the competitive inhibitory effect of β-sitosterol on NOX2 by exhibiting sustained binding inside the enzyme’s active region, indicating its ability to decrease NOX2-mediated ROS production. The antioxidant capabilities of these bioactive molecules enable them to scavenge ROS, alleviate oxidative stress, protect cellular macromolecules, and ultimately contribute to the preservation of liver function and structural integrity.

On the other hand, injection of UnSap in HCC-bearing mice led to significant upregulation of *TP53* and *BAX* gene expression, accompanied by downregulation of several other oncogenic and prosurvival genes. These effects are likely attributed to the bioactive constituents of UnSap, particularly phenolics and phytosterols, which have been previously reported to possess anti-inflammatory and anticancer properties^55,87,89,90^. These compounds serve as effective modulators of key cancer-related processes, including angiogenesis, metastasis, proliferation, and apoptosis^90,91^. The antioxidant activity of UnSap was evident in its ability to reduce intracellular free radical levels. This action led to suppression of NF-κB and its downstream targets, such as COX-2, BCL2, and AKT, while concurrently increasing the levels of the proapoptotic proteins, TP53 and BAX. These findings are consistent with previous reports^19,52^. In addition, the reduction in oxidative stress disrupted the stability of *HIF1A*, resulting in decreased expression of its associated genes, including *GLI1* and *BCL2*, and further elevation of *TP53* expression^83^. The antioxidant capacity of UnSap also contributed to the downregulation of key genes associated with cancer stemness and proliferation, such as *NOTCH1*, *PROM1*, *ALDH1A1*, *MYC*, and *ABCG1*. The downregulation of PROM1 gene expression indicates attenuation of cancer stem cell–associated traits, such as CD133, which is the functional regulator of HCC stemness. Previous studies showed that resveratrol induces apoptosis in HCC through multiple mechanisms, including *TP53* upregulation and activation of the caspase cascade^89^. In addition, resveratrol inhibits hepatic stellate cell–induced angiogenesis and reduces ROS production by downregulating *GLI1*^92^. Overall, the ability of UnSap to modulate these critical genes underscores its potential as a therapeutic agent for the treatment of HCC. These results confirmed the docking outcomes that predict the ability of UnSap constituents to target the key regulator proteins for HCC proliferation and stemness, including β-catenin, EGFR, NOX2, and SMO.

The current study further demonstrated that UnSap exhibited superior anti-HCC efficacy compared with that of 5-FU, significantly improving the evaluated biochemical and molecular parameters. Notably, 5-FU failed to downregulate the expression of *PROM1*, *ABCG1*, and *GLI1* in the liver tissues of HCC-bearing mice. The persistent overexpression of these genes is associated with the development of resistance to 5-FU therapy. These findings are consistent with those reported by Agnese et al*.*, who highlighted the role of these genes in mediating chemoresistance in HCC^93^.

Conversely, the administration of UnSap for six consecutive days in healthy mice led to a marked downregulation of *ALDH1A1*, *PROM1*, *NFKB1*, *PTGS2*, and *AKT1,* without adversely affecting other tested biochemical or molecular parameters. These effects are likely attributable to the phenolic and phytosterol constituents of UnSap, as previously reported by Tian et al*.*^94^. In contrast, the administration of 5-FU over the same period resulted in elevated serum levels of liver enzymes (ALT, AST, and ALP), increased TBARS concentrations, and upregulated the expression of *ABCG1* and *MYC*. Moreover, it decreased total serum protein and GSH levels and downregulated the expression of both *ALDH1A1* and *PTGS2*. These findings suggest that 5-FU administration induces oxidative stress and hepatic inflammation, even in nontumor-bearing animals. Histological examination of liver tissues from 5-FU–treated mice further confirmed hepatic inflammation, indicating potential hepatotoxicity. These observations are consistent with previous studies reporting liver toxicity as a side effect of 5-FU treatment^95^.

To advance UnSap into therapeutic applications, clinical translation involves the creation of standardized and reproducible formulations that provide constant phytochemical content and biological activity. Nanoemulsions, liposomal encapsulation, and biodegradable polymer-based delivery methods may improve the solubility, stability, and bioavailability of UnSap ingredients while reducing off-target toxicity. The extensive pharmacokinetic profiling, toxicity assessment, and dose-dependent investigations in relevant preclinical models are required to create safe and therapeutically translatable dosage regimens before early-phase clinical testing.

### Study limitations

Despite the promising findings of the present study, several limitations should be acknowledged. First, IPA-predicted pathways, including MAPK/ERK, 26S proteasome, and calpain signaling, remain unvalidated. Moreover, molecular docking predictions are inherently theoretical and require further biochemical and cellular validation. Second, the final group size for in vivo experiments was n = 7, adjusted from the initially allocated 10 animals due to model-associated attrition, and no formal a priori power analysis was performed. Third, the in vivo study was conducted using a single UnSap dose without dose–response evaluation. Fourth, apoptosis and mechanistic pathways were assessed primarily via caspase-3/7 activation and gene expression, without additional confirmatory assays, such as Annexin V–PI staining or protein-level analysis. Moreover, proteomic validation is required to confirm the transcriptional changes in the in vivo study. Fifth, the in vivo study used a short six-day treatment period; while significant improvements were observed in tumor histology and biochemical parameters, longer treatment and follow-up are required. Sixth, the lack of data on the bioavailability of UnSap warrants further pharmacokinetic and formulation-based investigations to increase its clinical potential.

## Conclusions

In summary, the current study investigated the anticancer potential of the UnSap fraction of BVVO, both in vitro and in vivo, in comparison with that of 5-FU. BVVO-UnSap is rich in phenolic and phytosterol compounds known for their antioxidant, anti-inflammatory, pro-apoptotic, and anticancer activities. These bioactive constituents were able to modulate key molecular targets, including proapoptotic and antiapoptotic genes, inflammatory mediators, cancer stem cell markers, and essential cell cycle regulators. They are also predicted to target key regulatory proteins that drive HCC proliferation and maintain stemness, including β-catenin, EGFR, NOX2, and SMO. Additionally, BVVO-UnSap effectively attenuated *p*-DAB-induced HCC in mice by restoring liver function, reducing oxidative stress, improving histological architecture, and regulating key tumor-associated genes. Collectively, these findings indicate that BVVO-UnSap could have potential relevance as a natural therapeutic approach for HCC.

## Supplementary Information

Supplementary Information 1.

Supplementary Information 2.

Supplementary Information 3.

Supplementary Information 4.

## Data Availability

All datasets produced or examined in this study are provided within the article.

## Abbreviations

ABCG1: ATP-binding cassette subfamily G member
AKT: Protein kinase B
ALDH1A1: Aldehyde dehydrogenase
ALP: Alkaline phosphatase
ALT: Alanine aminotransferase
ANOVA: One-way analysis of variance
Asc: Ascorbic acid
AST: Aspartate aminotransferase
BAX: BCL-2-associated X-protein
BCL2: B-cell lymphoma 2
MYC: Myelocytomatosis oncogene
CDKN1A: Cyclin-dependent kinase inhibitor 1
COX-2: Cyclooxygenase-2
EGFR: Epidermal growth factor receptor
p-DAB: P-dimethylaminobenzaldehyde
DMSO: Dimethyl sulfoxide
DPPH: Diphenyl –α-picrylhydrazyl
DTNB: 5,5'-Dithiobis-(2-nitrobenzoic acid)
E2F: E2 promoter binding factor
ERK: Extracellular signal-regulated kinase
FBS: Fetal bovine serum
5-FU: 5-Fluorouracil
γ-GGT: Gamma-glutamyl transferase
GLI1: Glioma-associated oncogene 1
GSH: Reduced glutathione
HIF1A: Hypoxia-inducible factor
HPLC: High-performance liquid chromatography
IACUC: Institutional Animal Care and Use Committee
JNK: C-Jun N-terminal kinase
KRAS: Kirsten rat sarcoma virus
MAPK: Mitogen-activated protein kinase
MTT: 3-(4,5-Dimethylthiazol-2yl-)-2,5-diphenyl tetrazolium bromide
NF-κB: Nuclear factor Kappa B
NOTCH1: Neurogenic locus notch homolog protein
PB: Phenobarbital
PBMCs: Peripheral blood mononuclear cells
PROM1: Prominin 1
PTGS2: Prostaglandin-Endoperoxide Synthase 2
RB1: Retinoblastoma 1
REC: Research Ethical Committee
RPMI-1640: Roswell Park Memorial Institute
SMO: Smoothened
TAC: Total antioxidant capacity
TBA: Thiobarbituric acid
TBARS: Thiobarbituric acid reactive substances
TMP: Tetramethoxy propane
UnSap: Unsaponifiable
VV: Vitis vinifera
