# Supplementary material for: Unsaponifiable fraction of black Vitis vinifera seed oil attenuates liver cancer progression by targeting apoptosis and key tumor-associated genes: In vitro, in vivo, and in silico studies
Source: Sci Rep. 2026 Apr 10;16:12018. doi: 10.1038/s41598-026-44404-9 (PMC13068968; doi:10.1038/s41598-026-44404-9)
Supplement: Supplementary file 3 — Supplementary Information 3. [file 41598_2026_44404_MOESM3_ESM.pdf]

Data File D:\DATA\PHENOLSFLAVONOIDS2019\FSQC909-1902699.D
Sample Name: FSQC909-19

===

Acq. Operator : FSQC Lab
Acq. Instrument : Instrument 1 Location : Vial 1
Injection Date : 11/11/2019 3:05:32 PM
Inj Volume : No inj
Acq. Method : C:\CHEM32\1\METHODS\PHENOLS AND FLAVONOIDS2019NEW_LC.M
Last changed : 11/11/2019 3:32:58 PM by FSQC Lab
(modified after loading)
Analysis Method : C:\CHEM32\1\METHODS\PHENOLS_CALIBRATION_11-2019_LC.M
Last changed : 11/12/2019 2:41:00 PM by FSQC Lab
(modified after loading)
Additional Info : Peak(s) manually integrated

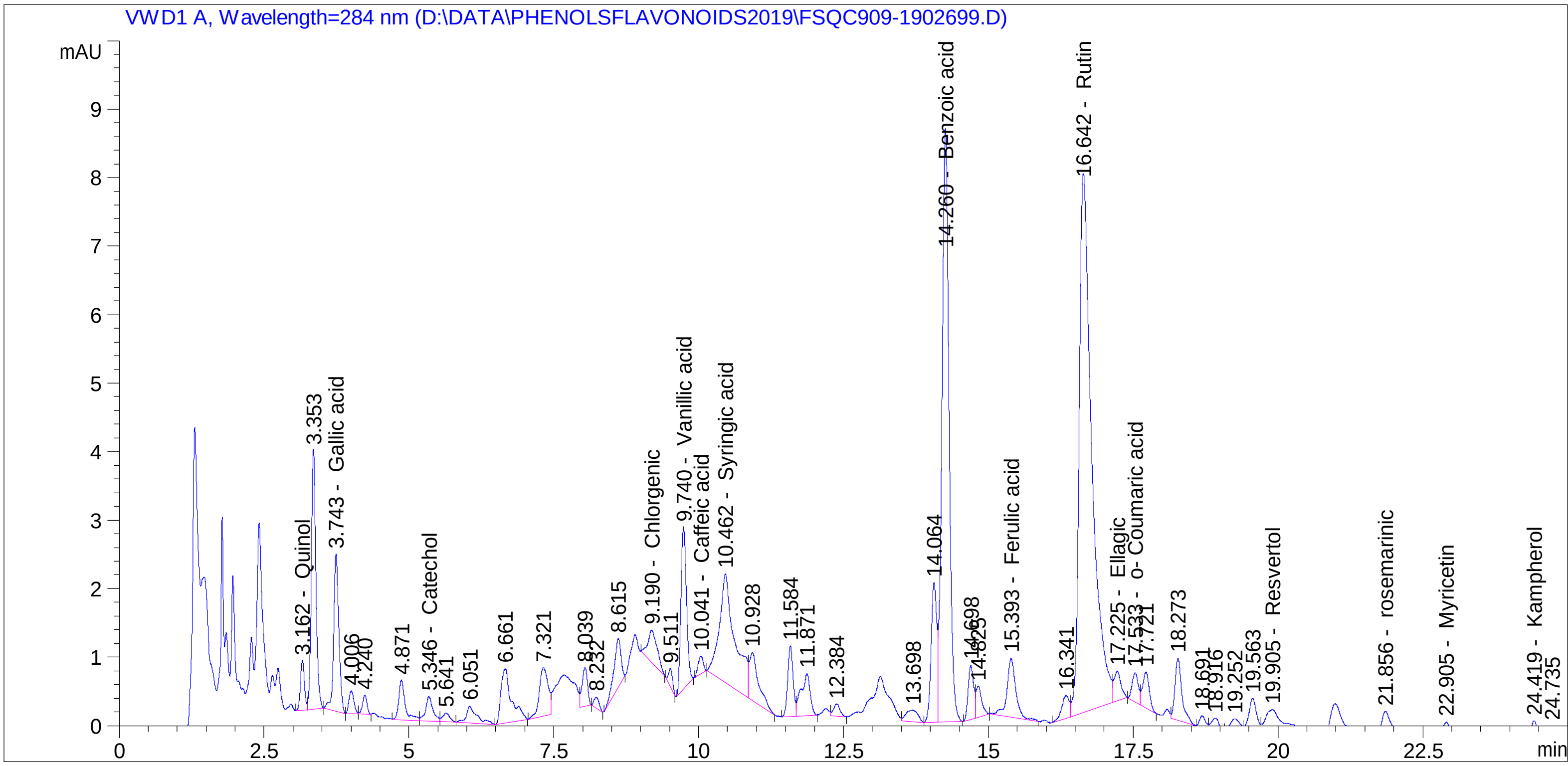

===

External Standard Report

===

Sorted By : Retention Time
Calib. Data Modified : 11/12/2019 2:25:23 PM
Multiplier: : 8.0000
Dilution: : 1.0000
Do not use Multiplier & Dilution Factor with ISTDs

Signal 1: VWD1 A, Wavelength=284 nm

RetTime [min]	Sig	Type	Area [mAU*s]	Amt/Area	Amount [mg/L]	Grp	Name
2.900	1		-	-	-		Pyrogallol
3.162	1	BV	3.44769	1.98246e-2	5.46792e-1		Quinol
3.743	1	BB	14.29075	7.43256e-3	8.49735e-1		Gallic acid

RetTime [min]	Sig	Type	Area [mAU*s]	Amt/Area	Amount [mg/L]	Grp	Name
5.346	1	VV	3.17539	2.71192e-2	6.88911e-1		Catechol
7.700	1		-	-	-		p- Hydroxy benzoic acid
8.900	1		-	-	-		Catchin
9.190	1	BBA	5.25953	7.05033e-3	2.96652e-1		Chlorgenic
9.740	1	BBA	15.70254	6.30904e-3	7.92543e-1		Vanillic acid
10.041	1	BB	1.55857	8.53809e-3	1.06458e-1		Caffeic acid
10.462	1	BV	29.89955	5.96486e-3	1.42677		Syringic acid
13.060	1		-	-	-		p- Coumaric acid
14.260	1	VB	73.97256	1.01552e-1	60.09661		Benzoic acid
15.393	1	BB	9.29126	5.42216e-3	4.03029e-1		Ferulic acid
16.642	1	VV	119.52937	3.50618e-2	33.52735		Rutin
17.225	1	VB	3.70827	2.97670e-3	8.83073e-2		Ellagic
17.533	1	BV	3.22823	1.94423e-2	5.02113e-1		o- Coumaric acid
19.905	1	VB	6.03580	2.83049e-2	1.36674		Resvertol
20.900	1		-	-	-		Cinnamic acid
21.600	1		-	-	-		Quercitin
21.856	1	BB	6.22212	1.48609e-1	7.39732		rosemarinic
22.200	1		-	-	-		Neringein
22.905	1	BB	3.68286	2.07394e-1	6.11043		Myricetin
24.419	1	VB	3.38213	7.17229e-2	1.94061		Kampherol

Totals : 116.14038

2 Warnings or Errors :

Warning : Calibration warnings (see calibration table listing)
Warning : Calibrated compound(s) not found

===

*** End of Report ***
