# Supplementary material for: Unsaponifiable fraction of black Vitis vinifera seed oil attenuates liver cancer progression by targeting apoptosis and key tumor-associated genes: In vitro, in vivo, and in silico studies
Source: Sci Rep. 2026 Apr 10;16:12018. doi: 10.1038/s41598-026-44404-9 (PMC13068968; doi:10.1038/s41598-026-44404-9)
Supplement: Supplementary file 4 — Supplementary Information 4. [file 41598_2026_44404_MOESM4_ESM.pdf]

Data File D:\DATA\STEROL_HYPERCOLNE\FSQC908-1902726.D
Sample Name: FSQC908-19

===

Acq. Operator : FSQC Lab
Acq. Instrument : Instrument 1 Location : Vial 1
Injection Date : 11/19/2019 2:12:00 PM
Inj Volume : No inj
Acq. Method : C:\CHEM32\1\METHODS\STEROLS FLUORESCENCE_HYPERCLONE.M
Last changed : 11/19/2019 2:09:54 PM by FSQC Lab
(modified after loading)
Analysis Method : C:\CHEM32\1\METHODS\STEROLS FLUORESCENCE_HYPERCOLNECALUV.M
Last changed : 12/12/2019 2:18:09 PM by FSQC Lab
(modified after loading)
Additional Info : Peak(s) manually integrated

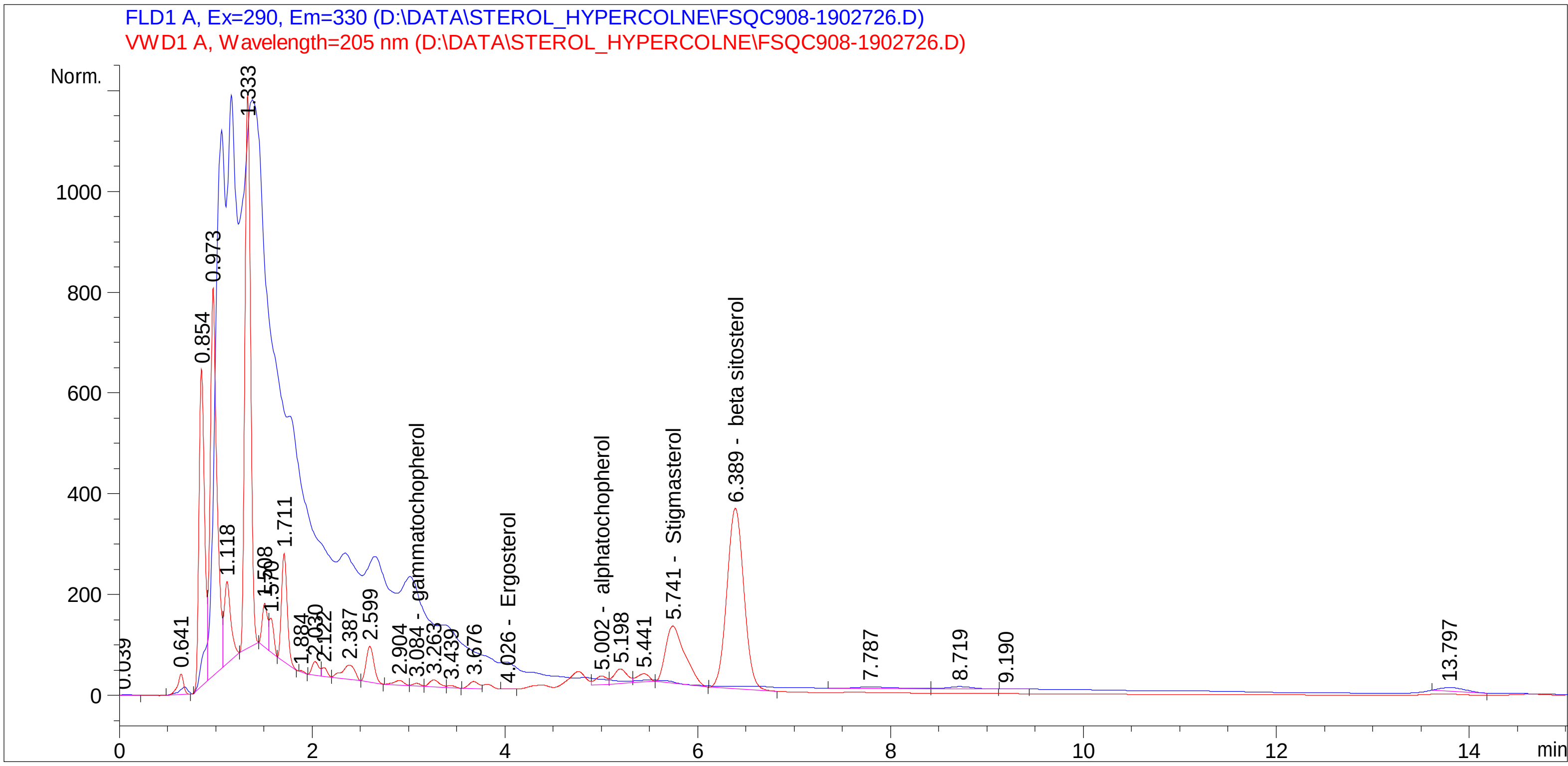

===

External Standard Report

===

Sorted By : Signal
Calib. Data Modified : 12/12/2019 1:59:29 PM
Multiplier: : 50.0000
Dilution: : 1.0000
Use Multiplier & Dilution Factor with ISTDs

Signal 2: VWD1 A, Wavelength=205 nm

RetTime [min]	Type	Area [mAU*s]	Amt/Area	Amount [mg/kg]	Grp	Name
3.084	VV	28.15591	2.08333e-2	29.32907		gammatochopherol
4.026	BB	3.08929	4.36305e-1	67.39365		Ergosterol
4.700		-	-	-		Cholesterol

RetTime [min]	Type	Area [mAU*s]	Amt/Area	Amount [mg/kg]	Grp	Name
5.002	VV	145.35574	4.41306e-1	3207.32002		alphatochopherol
5.741	BB	1566.41443	1.08900e-1	8529.16115		Stigmasterol
6.389	BB	4411.00928	4.01637e-1	8.85812e4		beta sitosterol

Totals : 1.00414e5

2 Warnings or Errors :

Warning : Calibration warnings (see calibration table listing)
Warning : Calibrated compound(s) not found

==

*** End of Report ***
